# Machine Learning for Radiomics in Oncology: Challenges, Limitations, and Future Directions

**DOI:** 10.3390/s26144619

**Published:** 2026-07-21

**Authors:** Rim Missaoui, Wajdi Saadaoui, Marco Del Coco, Abdelhamid Helali, Marco Leo, Pierluigi Carcagnì

**Affiliations:** 1 National High School of Engineering of Tunis (ENSIT), University of Tunis, 5 Rue Taha Hussein–Montfleury, Tunis 1008, Tunisia; 2 Laboratory of Micro-Optoelectronics and Nanostructures (LMON), University of Monastir, Avenue of the Environment, Monastir 5019, Tunisia; 3 ULR 2694 Metrics, Centre for Studies and Research in Medical Informatics (CERIM), University of Lille, 59000 Lille, France; 4 Institute of Applied Sciences and Intelligent Systems, National Research Council of Italy (CNR), 73100 Lecce, Italy

**Keywords:** oncology, radiomics, machine learning, deep learning, attention-based models, foundation models, self-supervised learning, acquisition variability, interpretability, clinical translation

## Abstract

In precision oncology, the combination of the strengths of both histopathology and medical imaging provides a fertile ground for tumor characterization. Although histopathology offers a definitive cellular diagnosis, this approach is invasive and only provides a small-scale characterization of the tumor, while medical imaging modalities, such as X-ray, CT, MRI, ultrasound, and PET scans, provide a complete characterization of the tumor but, until recently, relied on the subjective ability of a human observer. The application of machine learning to radiomics aims at filling this gap, as images are mined to reveal patterns of disease not visible to the naked eye. In this perspective paper, the trajectory of machine learning in radiomics for oncology applications is critically discussed. By exploring studies using different imaging modalities, we seek to look beyond the achievements of innovative algorithms and identify the systemic weaknesses in the field, which are holding it back from translating to the clinic. In this regard, we identify two major challenges in the field: the significant effects of inter-modality and inter-scanner variability in model generalizability, and the ‘interpretability gaps’ in understanding the rationale for the decision-making process in ML algorithms. In this paper, we assert that these challenges are holding back even the best of algorithms and thus set the direction for the field in the future, advocating for the development of ML systems with emphasis on their performance in real-world settings as opposed to the lab.

## 1. Introduction

In the era of precision oncology, the ability to decode the complex biological landscape of a tumor is essential for personalized treatment. Histopathology remains the gold standard (it provides a definitive, “microscopic”, diagnosis of cellular structures), but it is inherently limited by its invasive nature and its inability to capture the full spatial heterogeneity of a lesion from a single tissue fragment. On the other side, Medical Imaging (Radiology) is the non-invasive process of creating a visual representation of the body by using different acquisition modalities such as [1,2]:

- X-ray: Uses low-dose ionizing radiation to produce 2D images. It is the standard for breast cancer screening.
- Computed Tomography (CT): Uses rotating X-ray beams and computer algorithms to create detailed 2D slices that are reconstructed into a 3D view of the body. It is the primary tool for staging and monitoring lung or abdominal cancers.
- Magnetic Resonance Imaging (MRI): Uses powerful magnets and radio waves (no radiation) to align hydrogen protons in the body. MRI can be either 2D or 3D, depending on how the scan is acquired. In practice, many MRI exams are collected as a stack of 2D slices, and some are acquired as true 3D volumes that allow reconstruction in any plane. It offers the best soft tissue contrast, making it superior for imaging the brain, spine, and musculoskeletal system.
- Ultrasound: Uses high-frequency sound waves to visualize tissues in real-time. It is often used for initial screenings of the liver, thyroid, and breasts.
- Positron Emission Tomography (PET): Involves injecting a radioactive tracer (e.g., FDG, a sugar molecule) that accumulates in metabolically active cancer cells, appearing as “bright spots”. As with MRI, PET can operate in either 2D or 3D acquisition modes, but it is fundamentally a 3D imaging technique because it measures tracer distribution throughout the body volume.

Each of these imaging modalities relies on distinct sensor technologies that convert physical signals into digital images. X-ray and CT depend on solid-state or scintillator-based detectors that capture transmitted photons; MRI utilizes radiofrequency coils as electromagnetic sensors to detect hydrogen proton relaxation signals; PET employs scintillation crystals coupled with photomultiplier tubes or silicon photomultipliers (SiPMs) to detect annihilation photons from radiotracers; and ultrasound uses piezoelectric transducers that convert mechanical acoustic waves into electrical signals. Advances in sensor technology—including photon-counting detectors for CT, silicon photomultipliers for PET, and novel coil designs for MRI—are enabling higher spatial resolution, lower radiation doses, and new functional imaging capabilities. These sensor-level innovations directly impact radiomics by altering the signal-to-noise characteristics, spatial resolution, and texture properties of the acquired images, which in turn affects the reproducibility and generalizability of machine learning models—a theme we return to in Section 4.1.

**Figure 1:**
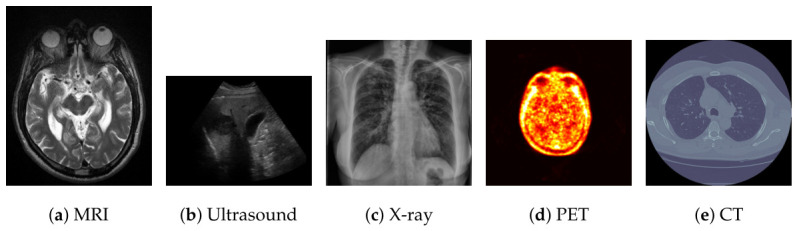
Representative examples of medical imaging modalities commonly used in precision oncology and radiomics: (**a**) Magnetic Resonance Imaging (MRI), (**b**) Ultrasound (US), (**c**) Chest X-ray (CXR), (**d**) Positron Emission Tomography (PET), and (**e**) Computed Tomography (CT). Representative images were obtained from publicly available datasets [3,4,5,6,7].

Figure 1 presents representative examples of the principal medical imaging modalities commonly used in precision oncology and radiomics.

Medical imaging remains a tool that fundamentally relies on human experts to visually interpret and translate these complex pictures into a clinical diagnosis. Radiomics is the process of automatically converting medical images into quantitative (measurable) data, operating on the premise that pixels contain a wealth of hidden information that far exceeds the limits of human visual interpretation [8]. Any disease that changes the texture, density, or shape of tissue—even if those changes are too subtle for the human eye—is a candidate for radiomics. Oncology serves as the premier domain for radiomics because it provides a “data-rich” environment where high-stakes clinical decisions require more than just visual inspection. In recent years, machine learning (ML) has fundamentally reshaped radiomics for tumor detection, classification, and segmentation across modalities [1]. In this perspective paper, we provide a critical look at the trajectory of machine learning in radiomics for oncology, analyzing its rise and the significant hurdles that still block its path to the clinic. By reviewing tumor studies across multiple imaging modalities, we expose how acquisition variability and ‘interpretability gaps’ undermine even the most advanced models. Rather than cataloging individual architectures, we identify the systemic flaws in current research and set a future direction for developing ML systems that are not just accurate in the lab but reliable in the hospital [9,10]. The rest of the paper is organized as follows: Section 2 describes the study selection, whereas Section 3 describes the role of ML in radiomics for oncology. Section 4 then describes the ongoing challenge in extending predictive models from laboratory outcomes to real-world clinical support systems. How recent studies in machine learning can address the mentioned drawbacks is the core of the discussion in Section 5. Section 6 discusses future directions for improving the clinical robustness of ML-based radiomics. Finally, Section 7 concludes the paper.

## 2. Study Selection

**Figure 2:**
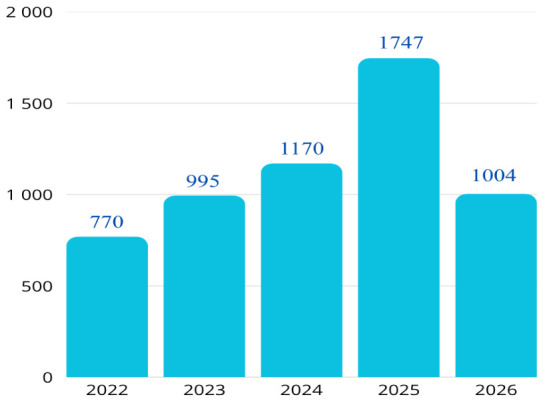
Annual number of Scopus-indexed publications related to machine learning for radiomics in oncology from 2022 to 2026.

The primary aim of this paper is to provide a critical synthesis, conceptual framing, and forward-looking roadmap for the application of machine learning in radiomics for precision oncology, rather than an exhaustive enumeration of all published studies. Accordingly, the literature cited throughout this manuscript was selected to illustrate key trends, methodological transitions, and persistent challenges, rather than to serve as a comprehensive state-of-the-art review. As this manuscript is a perspective article, it does not follow a systematic review methodology or the PRISMA reporting guidelines. Rather, the selected literature provides representative examples that support the critical discussion, conceptual synthesis, and future directions presented in this paper. The literature selection was performed through searches in PubMed, Google Scholar, Scopus, and Web of Science, using combinations of the following general search terms: “radiomics”, “machine learning”, “deep learning”, “convolutional neural network”, “attention mechanism”, “transformer”, “multimodal learning”, “foundation model”, “self-supervised learning”, “oncology”, “cancer”, “CT”, “MRI”, “PET”, and “ultrasound”. We prioritized peer-reviewed original research articles, major conference proceedings (e.g., MICCAI, RSNA, NeurIPS), and high-impact review articles. For claims about the current state of the field, we gave priority to literature published from 2022 onward. Older studies (pre-2022) were retained only as foundational background. As shown in Figure 2, the number of Scopus-indexed publications related to machine learning for radiomics in oncology has increased markedly over recent years, highlighting the rapid expansion of this research field. This trend supports our decision to prioritize studies published from 2022 onward when discussing the current state of the art. The value reported for 2026 corresponds to a partial year, as the literature search was conducted in July 2026.

The studies included in the summary tables in Section 3 were selected as exemplary illustrations of major methodological families and clinical applications. The selection criteria were: (i) methodological innovation (first application of a technique, novel architecture, or fusion strategy); (ii) clinical relevance (external validation, multicenter data, clearly defined clinical endpoint); (iii) diversity (coverage of different cancer types, imaging modalities, and ML families); (iv) recency (priority given to studies from 2022–2025 for current-state examples); and (v) field influence (high citation count or publication in top-tier journals). These tables are not intended as comprehensive systematic reviews of each subfield, but rather as illustrative snapshots that support the critical discussion in the main text. We acknowledge that the studies summarized in the tables address heterogeneous clinical tasks—including classification, segmentation, survival prediction, molecular marker prediction, and treatment response—and report different performance metrics (e.g., AUC, accuracy, Dice coefficient, C-index). Direct quantitative comparison across these studies is not meaningful, and the tables are not intended to imply any ranking or superiority of one method over another. Rather, they serve to illustrate the range of methodological approaches and clinical applications that characterize each ML paradigm. A future systematic review or meta-analysis would be valuable to quantitatively synthesize performance metrics across studies, but such an effort lies beyond the scope of the present Perspective. Besides, the summary tables do not include all methodological details that would be required for a systematic quality assessment (e.g., data split strategy, confidence intervals, class balance, segmentation type, or code/data availability). Such detailed extraction is beyond the scope of a Perspective article and would require a full systematic review with standardized data extraction protocols. However, to provide essential context for interpreting the reported results, we have included sample size and external validation status as two key indicators of study quality and generalizability. Readers should note that reporting practices vary considerably across the literature, and not all studies reported all items. We refer readers to the original publications for complete methodological details and encourage the community to adopt more standardized reporting guidelines (e.g., TRIPOD-AI, DECIDE-AI) to facilitate future meta-analyses.

## 3. The Role of ML in Radiomics for Oncology

Machine learning has not merely influenced radiomics—it has redefined its methodological foundations while simultaneously exposing its structural limitations. The field has shifted from expert-defined, hand-crafted features toward methods that automatically learn complex imaging patterns linked to tumor biology, treatment response, and patient outcomes. Yet this transition did not occur as a clean paradigm shift. It unfolded as a series of methodological responses to unresolved problems in earlier approaches—each generation inheriting the core problems of its predecessor in a new and less transparent form. What has remained constant throughout—and what the field has been slow to confront—is the persistent gap between controlled performance and clinical reliability. Two challenges define this gap: acquisition variability, which undermines the biological meaning of learned representations, and interpretability gaps, which prevent clinicians from assessing whether model decisions can be trusted.

**Figure 3:**
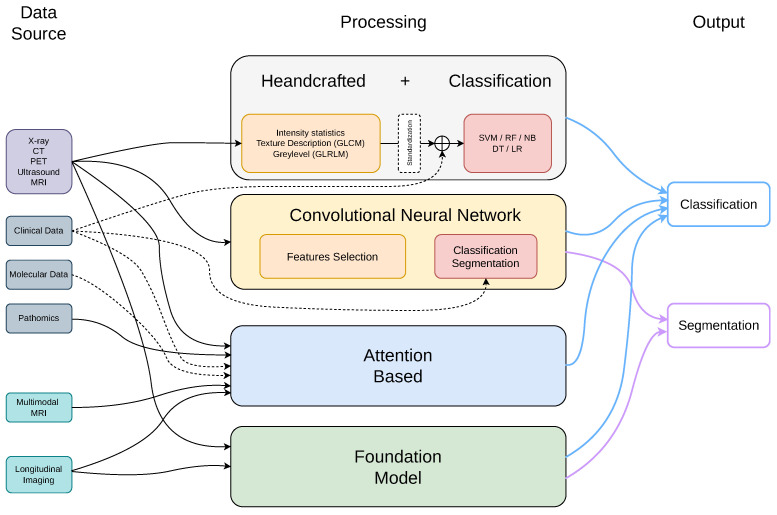
Schematic overview of the analysis strategies, encompassing input types, processing approaches, and output tasks. The purple boxes in the data source column represent the most common acquisition modalities, the grey boxes indicate non-image auxiliary data, and the light cyan boxes denote advanced imaging techniques that exploit additional dimensions. In the processing column, colors are primarily used to emphasize different methodological families, with the exception of the orange and red boxes, which specifically highlight the feature management phase (light orange) and the classification/segmentation step (light red), respectively. Finally, the output column illustrates the possible flows toward classification (light blue) or segmentation (mauve). Dotted lines indicate optional data or processing stages.

The schema in Figure 3 provides a schematic overview of the analysis strategies discussed in this section. Specifically, the right-hand side depicts the range of potential inputs, spanning standard imaging data, additional contextual data, and advanced imaging modalities. The processing pipeline is organized from top to bottom according to increasing methodological innovation and complexity. Solid arrows denote alternative standard inputs when multiple options are available, whereas dotted arrows indicate optional supplementary data incorporated in specific data-fusion strategies. The right-hand side further illustrates how the different processing approaches may be oriented toward either classification or segmentation outputs. The schema reports the most investigated approaches, while not excluding other possible data integrations and pipeline configurations.

### 3.1. Traditional Machine Learning and Hand-Crafted Radiomics

The hand-crafted radiomics paradigm emerged in the mid-2000s and gained significant momentum through the 2010s, as researchers began systematically extracting quantitative descriptors from segmented regions of interest and linking them to clinical outcomes through classical machine learning models [8,11]. This approach rested on three major families of features: intensity statistics, which characterize the distribution of voxel values within a region; texture descriptors derived from gray-level co-occurrence matrices (GLCM) and gray-level run-length matrices (GLRLM), which encode spatial relationships between voxel intensities; and shape metrics describing lesion geometry. Support Vector Machines (SVM), Random Forests (RF), Naïve Bayes (NB), Decision Tree (DT), Logistic Regression (LR), K-Nearest Neighbors (KNN) and gradient boosting methods, such as eXtreme Gradient Boosting (XGBOOST) and Light Gradient Boosting Machine (LightGBM), constituted the dominant classifiers of this era and were applied across a broad range of oncologic tasks, including tumor classification, treatment response prediction, and survival analysis [12]. This framework established radiomics as a systematic approach to quantitative decision support and generated early enthusiasm for its potential to complement—and eventually reduce reliance on—subjective radiological interpretation.

Early studies focused primarily on establishing the feasibility of SVM- and RF-based radiomic classifiers for standard oncologic imaging tasks. Parmar et al. (2015) [11] investigated conventional machine learning techniques for radiomics-based survival prediction in non-small cell lung cancer (NSCLC), using pre-treatment CT scans from two different cohorts (464 patients total). A total of 440 hand-crafted radiomic features were extracted and evaluated using 14 feature selection methods and 12 classifiers. The combination of Wilcoxon feature selection and the Random Forest classifier achieved the best performance (AUC ≈ 66.0%) and stability for overall survival prediction. Zheng et al. (2022) [13] suggested a CT-based radiomics framework for benign and malignant parotid tumor differentiation based on multiphase CT scans from 388 patients. A total of 2874 hand-crafted radiomic features were extracted and evaluated with LR, SVM, and RF classifiers. The proposed model achieved the best and most stable performance in the test cohort using the SVM classifier (AUC = 84.0%). Furthermore, a combined model integrating radiomics and clinical-radiological features achieved the highest diagnostic performance (AUC = 85.4%). Similarly, Li et al. (2024) [14] evaluated several radiomics-based machine learning classifiers for predicting pathological complete response (pCR) to neoadjuvant therapy in breast cancer using pre- and post-contrast T1-weighted MRI images from the Duke-Breast-Cancer-MRI dataset (281 patients). A total of 529 hand-crafted radiomic features were analyzed using SVM, RF, DT, KNN, XGBoost, and LightGBM classifiers. The highest overall performance was achieved with the LightGBM classifier, with an accuracy of 74.0% and an AUC of 82.3%, while RF performed best for luminal breast cancer, achieving an accuracy of 87.0% and an AUC of 91.4%.

For non-small cell lung cancer (NSCLC), Tang et al. (2023) [15] compared several classic machine learning and deep learning methods for prognosis prediction using CT-based radiomic features from The Cancer Imaging Archive (TCIA) dataset [16]. A total of 107 hand-crafted radiomic features were extracted from 394 patients and analyzed using DT, Boosted Tree (BT), RF, SVM, Generalized Linear Model (GLM), and deep learning artificial neural networks (DL-ANNs). RF achieved the best overall predictive performance with an AUC of 93.8%, outperforming both traditional classifiers and DL-ANNs. Ma et al. (2024) [17] developed a multimodal radiomics framework based on multiphase CT and MRI images from 221 patients to distinguish hepatocellular carcinoma (HCC) from non-HCC liver lesions. The combined CT+MR model with the SVM classifier achieved the best diagnostic performance, with an accuracy of 82.4.

Shayesteh et al. (2021) [18] investigated MRI-based pre-, post-, and delta-radiomic features alongside machine learning algorithms to predict treatment outcome in locally advanced rectal cancer (LARC) patients following neoadjuvant chemoradiotherapy (nCRT). Using T2-weighted MRI scans from 53 patients, 96 hand-crafted radiomic features were extracted and analyzed with KNN, NB, RF, and XGBoost classifiers, where the highest predictive performance was achieved with the delta-radiomic RF model (AUC = 96%). Xie et al. (2025) [19] developed MRI-based radiomics models to distinguish the primary origin of brain metastases based on contrast-enhanced T1-weighted MRI images from 180 patients. The best diagnostic performance was achieved using the LightGBM model with AUCs of 87.5% and 86.6% in the training and validation sets.

Beyond individual studies, several broader reviews have examined the overall trajectory and limitations of classical radiomics pipelines. Tabassum et al. [20] reviewed the application of radiomics and classical ML to brain tumor habitat characterization across multiple MRI modalities, highlighting both the broad applicability of traditional pipelines and the persistent methodological heterogeneity that limits reproducibility across studies. Rogers et al. [8] formalized the conceptual shift this paradigm represented—from qualitative visual interpretation to systematic quantitative imaging analysis—and outlined the standardization challenges that remained unresolved at the time. Piedimonte et al. [21] further reviewed the use of classical ML and radiomics for treatment response and survival prediction in advanced ovarian cancer, noting that while promising results were reported across multiple studies, small cohort sizes and the absence of prospective external validation remained common limitations across the field.

Despite these advances, the hand-crafted radiomics paradigm relied on assumptions whose limitations became progressively apparent as studies expanded toward multicenter and real-world settings. The approach assumed that tumor phenotype could be adequately represented by a fixed vocabulary of intensity, texture, and shape descriptors and that these features would remain stable across patients, scanners, and acquisition protocols. In practice, radiomic features proved highly sensitive to imaging parameters unrelated to tumor biology, including tube voltage and current in CT, magnetic field strength and pulse sequences in MRI, slice thickness, reconstruction kernels, and contrast administration protocols [9,10]. Zhao [9] systematically reviewed these sources of instability and reported that first-order intensity and higher-order texture features were particularly susceptible to acquisition-related variability, whereas shape features generally showed greater reproducibility across protocols. As highlighted in studies such as [17,19], feature stability and harmonization across imaging centers remained persistent challenges even when strong performance was achieved on internal validation datasets.

To address these limitations, several methodological strategies were introduced to improve reproducibility and standardization. The Image Biomarker Standardization Initiative (IBSI) established standardized feature definitions to reduce inter-pipeline variability [22], while ComBat harmonization—adapted by Orlhac et al. [23] from genomic batch correction methods—was introduced to reduce scanner-related effects in radiomic feature distributions. Test–retest imaging [24] and perturbation analysis [25] further provided complementary frameworks for identifying reproducible features before model training. Although these approaches improved reproducibility assessment and feature standardization, acquisition-dependent variability remained a persistent limitation across imaging centers. Consequently, while hand-crafted radiomic features offered greater interpretability than later deep learning representations, their clinical utility remained constrained by challenges related to robustness, reproducibility, and external generalizability [26,27].

The traditional machine learning era established the conceptual and methodological foundations of radiomics by demonstrating that quantitative imaging features could provide clinically relevant information across multiple oncologic applications. At the same time, the limitations associated with fixed feature spaces, acquisition-dependent representations, and pipeline variability progressively motivated the transition toward data-driven approaches capable of learning imaging representations directly from raw data. This development would define the next phase of the field’s evolution. Table 1 summarizes representative studies using traditional machine learning and hand-crafted radiomic features in oncologic imaging.

### 3.2. Convolutional Neural Networks: From Feature Engineering to End-to-End Learning

The move toward convolutional neural networks was, in large part, a response to what classical radiomics could not do. Hand-crafted features were interpretable and computationally tractable, but they depended on assumptions about biological stability that multicenter studies kept failing to confirm. CNNs offered a different starting point: instead of defining what to measure, the network learns from the data itself, building hierarchical representations that capture spatial patterns at multiple scales without manual intervention [2,28]. Architectures like U-Net, ResNet, VGG, DenseNet, and InceptionV3 became the standard tools of this era, covering tasks from tumor segmentation and detection to grading, mutation prediction, and treatment response assessment [2,29]. The shift was significant—not just technically, but conceptually. It moved oncologic imaging analysis away from the question of which features to extract and toward the question of how much can be learned directly from the image.

The earliest CNN studies in this space were largely focused on showing that learned features could match or beat hand-crafted ones on standard tasks. Mahmud et al. (2023) [30] tested ResNet50, VGG16, InceptionV3, and a custom CNN on 3264 brain MRI images for tumor detection. The custom architecture came out ahead, reaching an accuracy of 93.3% and an AUC of 98.43%. For tumor grading, Yang et al. (2025) [31] developed BrainCNN, comparing it against MobileNet, InceptionV3, and ResNet50. Their model reached 99.45% accuracy in separating low-grade from high-grade tumors. In lung cancer, Zhang et al. (2024) [32] used a 3D-CNN to predict EGFR mutation status from CT scans across 660 patients at two centers, achieving an AUC of 94.7%, outperforming both radiomic and clinical models.

Segmentation was where CNN architectures arguably had their most consistent clinical impact. Chaudhary et al. (2026) [33] compared U-Net and SSD for brain tumor segmentation on MRI—U-Net reached 97.73% accuracy, while SSD struggled at 58%. Yu et al. (2025) [34] compared RNN, U-Net, WFCM, and SNAKE against manual radiologist segmentation on CT data from 1429 lung cancer patients. The RNN method achieved a Dice of 0.803 and held up well across downstream diagnostic tasks. Devadas et al. (2026) [35] combined Mask R-CNN segmentation with radiomic and GLCM texture analysis for MRI-based brain tumor detection, achieving 98.15% classification accuracy and 98.98% localization accuracy.

Once the basic viability of CNN-based radiomics was established, the field naturally moved toward integration—combining deep features with classical radiomic descriptors or clinical data to build richer predictive models. Chen et al. (2023) [36] built a nomogram for axillary lymph node metastasis prediction in breast cancer, achieving a training AUC of 80% and a testing AUC of 71%—a gap that reflects the generalization challenge common to this type of model. Khanfari et al. (2023) [37] combined CNN-derived deep features with classical radiomics from multiparametric prostate MRI, achieving an AUC of 94% for Gleason grading. Qureshi et al. (2023) [38] fused Grey Level Co-occurrence Matrix (GLCM), Histogram of Oriented Gradients (HOG), and Local Binary Patterns (LBP) features with CNN latent representations to predict $O^{6}$-methylguanine-DNA methyltransferase (MGMT) methylation status on the 2021 RSNA Brain Tumor Radiogenomic Classification dataset (BraTS-2021), reporting an accuracy of 96.84%. Cho et al. (2024) [39] studied brain metastasis response to stereotactic radiosurgery using sequential MRI from 194 patients; the Conv-GRU achieved AUCs of 87.8% and 83.4% on developmental and external temporal test sets.

Several studies and reviews have summarized the broader development of CNN-based oncologic imaging. Jiang et al. [2] covered detection, segmentation, and outcome prediction across modalities, noting consistent benchmark gains alongside persistent questions about real-world generalizability. Gao et al. [29] reviewed deep learning in breast cancer imaging specifically, identifying deployment robustness as the field’s central unresolved problem. Salmanpour et al. [40] compared hand-crafted and deep radiomic approaches head-to-head across PET and Single Photon Emission Computed Tomography (SPECT) tasks, offering one of the more rigorous direct evaluations of the two paradigms. CNNs clearly moved the field forward, with consistent performance gains reported across multiple cancer types and imaging modalities. Recent deep learning applications on chest X-rays for lung cancer detection and comparative studies demonstrating the efficacy of deep features over hand-crafted radiomics on planar X-ray data.

Despite these advances, these models come with their own constraints. They need large annotated datasets, which are not easy to assemble in oncology [41,42]. When training data are limited or not representative enough, models can latch onto dataset-specific patterns that do not carry over to new institutions or acquisition settings [40]. Interpretability also becomes harder as architecture depth increases—CNN representations live in high-dimensional spaces that are not directly readable, and while tools like Gradient-weighted Class Activation Mapping (Grad-CAM) and SHapley Additive exPlanations (SHAP) help, their outputs are sensitive to small input changes [43,44]. These gaps—in generalizability, in transparency, and in the ability to reason across long spatial ranges—are what pushed the field toward attention mechanisms and transformer-based designs. Table 2 summarizes representative studies using CNN-based deep learning radiomics in oncologic imaging.

### 3.3. Attention Mechanisms and Multimodal Learning

Standard CNNs analyze images using fixed spatial hierarchies, excelling at identifying local patterns. In contrast, they have no built-in mechanism to relate distant regions of an image to one another or to dynamically adjust which areas matter most depending on the clinical question. Transformers addressed this through self-attention, which allows each input element to be weighted relative to all others, giving the model a way to prioritize diagnostically relevant regions without being constrained by fixed receptive fields [45,46,47]. In oncologic imaging, this distinction matters—tumor boundaries, peritumoral tissue, and spatial relationships between structures all carry information that local convolution alone can miss. At the same time, clinical decision-making draws on more than imaging: molecular profiles, treatment history, laboratory values, and clinical context all contribute to how a case is assessed. Single-modality models cannot incorporate this, and multimodal frameworks emerged specifically to bridge that gap, combining CT, MRI, PET, genomics, and clinical variables within unified architectures [48,49]. Together, these two developments—attention mechanisms and multimodal integration—defined a new phase in the field.

Early attention-enhanced studies focused on demonstrating that self-attention could improve upon standard CNN fusion strategies. Zhou et al. (2023) [50] applied channel-attention fusion to multiparametric MRI for predicting neoadjuvant chemoradiotherapy response in rectal cancer, using T2-weighted, T1-contrast-enhanced, and apparent diffusion coefficient (ADC) map sequences from 422 patients across two hospitals. Their attention-enhanced model achieved AUCs of 89.8% and 87.3% on internal and external validation, outperforming standard concatenation-based fusion approaches.

As attention-based architectures became more established, the field increasingly moved toward integrating imaging with clinical and molecular data within multimodal frameworks. Nishizawa et al. (2025) [51] built an attention-based framework for predicting pathological complete response in breast cancer, combining longitudinal whole-breast MRI with clinical variables through 3D CNNs and self-attention modules. Evaluated on the multi-institutional I-SPY datasets, the model reached AUCs of 73% and 71% on internal and external cohorts, outperforming both imaging-only and clinical-only baselines. Zhang et al. (2025) [52] proposed the multimodal full information feature fusion transformer (MuFi), integrating whole-slide pathology images, MRI, and clinical data through a hierarchical vision transformer with a radiology-guided co-attention mechanism. Across 567 patients at two institutions, the model achieved AUCs of 90.2%, 81.8%, and 81.6% on training, validation, and external testing. Sarwar et al. (2025) [53] introduced a Multimodal Deep Learning framework with a Cross-Attention mechanism (MDL-CA), pairing Graph Attention Networks for genomic representation with 3D DenseNet imaging features through cross-modal attention for brain cancer diagnosis, achieving accuracies of 96.22–98.46% across four benchmark datasets.

Several studies pushed further by building transformer-based pipelines that incorporated radiomics, pathomics, and longitudinal imaging together. Gan et al. (2025) [54] fused multi-temporal CT radiomics, whole-slide pathomics, and longitudinal imaging data through transformer attention to predict major pathological response in NSCLC patients undergoing neoadjuvant immunochemotherapy. Across multicenter cohorts of 271 patients, the model reached an external AUC of 85.8%, outperforming both radiomics-only and pathomics-only baselines while also improving survival stratification. Ji et al. (2026) [55] combined radiomic descriptors with Vision Transformer embeddings from ultrasound images in a TabTransformer framework for ovarian tumor analysis, drawing on 3156 patients across eight institutions, achieving a training AUC of 98% and an external AUC of 95.8%.

Recent review studies have highlighted the growing importance of multimodal artificial intelligence in precision oncology. Zhang et al. (2025) [56] reviewed multimodal integration strategies combining clinical, imaging, multi-omics, and electronic health record data, emphasizing that AI-driven fusion methods can enhance diagnosis, prognosis, biomarker discovery, and treatment-response prediction, while challenges such as data heterogeneity, missing modalities, harmonization, and computational complexity remain unresolved. Similarly, Yang et al. (2025) [57] surveyed 651 studies on multimodal deep learning for precision oncology and reported that cross-modal fusion approaches consistently outperform unimodal systems. Al-Zoghby et al. (2025) [49] reviewed recent multimodal deep learning architectures involving radiology, histopathology, genomics, and clinical data, highlighting the effectiveness of transformer-based, attention-driven, and graph-based fusion methods while noting that cross-modal alignment, scalability, explainability, missing data, and clinical translation still limit widespread adoption.

Despite these advances, several practical challenges remain, particularly because multimodal learning assumes that imaging, genomic, and clinical data are complementary and consistently available—an assumption that is difficult to satisfy in routine clinical practice [53,58]. Missing modalities and modality imbalance remain common, particularly when one data source dominates predictive performance [49]. Fusion strategies also involve important tradeoffs: early fusion requires uniform data availability, and late fusion preserves modularity but may miss cross-modal interactions, while cross-attention mechanisms are more expressive but often harder to interpret.

Handling missing modalities remains one of the most consequential, yet underexplored, challenges in multimodal learning for clinical applications. Most research assumes that imaging, genomics, histopathology, laboratory values, and longitudinal follow-up data are available for every patient, but real clinical environments rarely meet that assumption. Tests are skipped because of cost or contraindications, facilities capable of running certain assays are not always accessible, older records are frequently incomplete, and institutions differ in which diagnostic workflows they follow. These gaps carry several consequences for clinical translation. The first concern is applicability: a model trained exclusively on complete multimodal data cannot be reliably deployed when a modality is missing, unless it was explicitly designed to accommodate that scenario. Closely related is equity, since patients treated in resource-limited settings are disproportionately likely to have incomplete records; a model unable to function without full data effectively excludes this population, an outcome that risks deepening rather than reducing existing health disparities. Validation is affected as well, because accuracy measured on complete-data test sets tends to overstate how a model will perform once exposed to the incomplete data typical of routine care. Finally, robustness suffers when models become implicitly dependent on a modality that, in practice, is not consistently available. Several methodological responses exist to address these gaps. Imputation approaches—mean substitution, regression-based estimation, and generative modeling—are among the most established, while modality dropout during training offers another route, encouraging models to learn representations that do not hinge on any single input. A further direction lies in modality-agnostic architectures designed to operate across varying combinations of available inputs [49,58]. Despite this promise, none of these strategies has been widely adopted within radiomics or multimodal medical AI more broadly, and how well they perform in real clinical settings remains an active area of investigation.

Interpretability also remains an open question in multimodal AI. Attention weights were initially viewed as a transparency mechanism, yet Chung et al. [59] showed that attention maps in transformer-based medical imaging models do not consistently correspond to clinically meaningful regions, suggesting they reflect internal computational weighting rather than causal reasoning. In multimodal systems, understanding how each modality contributes to predictions remains equally challenging [60]. Although methods such as Grad-CAM and SHAP provide useful qualitative insights, their outputs can vary substantially with small input perturbations and should therefore be interpreted cautiously [51,61].

Attention-based and multimodal architectures meaningfully expanded what models can represent in oncologic imaging. Integrating more data types does introduce more sources of variability and makes thorough validation more demanding—not reasons to avoid multimodal learning, but practical considerations for how these models are evaluated before clinical use. Addressing them at scale is part of what motivated the next shift in the field toward foundation models and self-supervised learning, which aimed to learn general-purpose representations from large unlabeled datasets, although this transition also introduced new methodological and clinical challenges [62,63]. Table 3 summarizes representative studies using attention mechanisms and multimodal learning in oncologic imaging.

### 3.4. Foundation Models and Self-Supervised Learning (SSL)

Earlier generations of oncologic imaging models shared a common bottleneck: the need for large quantities of labeled data. Annotating medical images at scale is expensive, slow, and requires clinical expertise that is not always available. Foundation models and self-supervised learning emerged as a direct response to this constraint. Rather than training on labeled examples from scratch, these approaches pretrain on large collections of unlabeled imaging data using objectives such as contrastive learning, masked image reconstruction, and self-distillation, then adapt the learned representations to downstream tasks with minimal supervision [64,65]. Foundation models offer cross-task transferability and representation reuse at a scale that task-specific supervised models cannot match.

Early work established the data efficiency advantages of SSL-pretrained representations across oncologic imaging tasks. Pai et al. (2024) [66] showed that a foundation model pretrained on 11,467 CT lesions outperformed supervised and pretrained baselines, achieving AUCs of 94.4% for lung nodule malignancy prediction and 63.8%/65.3% for 2-year NSCLC survival prediction on two external cohorts. Chen et al. (2024) [67] introduced UNI, a self-supervised ViT-Large model pretrained on over 100 million Hematoxylin and Eosin histopathology patches from 100,426 whole-slide images, achieving 97.6% AUC on 43-class cancer-type classification and improving average performance across 15 slide-level tasks by 8.3–10.0% over strong pathology-pretrained baselines. Huang et al. (2026) [68] proposed BSTNet, a self-supervised temporal learning framework for longitudinal breast MRI, achieving AUCs of 88.2%, 85.7%, and 85.4% across internal and two external validation cohorts. Missaoui et al. (2026) [69] demonstrated that DINO vision transformer features used as frozen extractors with lightweight classifiers could achieve competitive results in brain tumor classification—reaching 98.17% accuracy on a 15-class MRI dataset and 99.08% on a 4-class dataset—offering a practical deployment path in computationally constrained settings.

Among SSL objectives, contrastive learning produced several strong results in oncologic imaging. Renugadevi et al. (2025) [70] combined SimCLR-based contrastive learning with nnU-Net deep features, hand-crafted radiomics, and clinical variables for glioblastoma survival prediction on multimodal MRI, achieving C-index values of 0.87 (internal) and 0.86 (external). Li et al. (2026) [71] proposed the Unified CT-Based Lung Cancer Imaging Foundation (UCLIF) model, combining contrastive masked image modeling with Vision Transformers pretrained on 33,901 unlabeled chest CT scans, achieving AUCs of up to 97% for survival prediction and 96% for lung cancer classification across multicenter datasets.

Masked autoencoding approaches formed a second major branch, with several studies demonstrating strong cross-task and cross-modality transferability. Yang et al. (2025) [72] proposed CRCFound, a foundation model pretrained on 5137 unlabeled 3D CT volumes for colorectal cancer, achieving AUCs of 88.9–95.2% across TNM staging, MSI prediction, and survival analysis. Li et al. (2026) [73] proposed the Unified Multimodal Brain Imaging Foundation (UMBIF) model, pretrained on 51,029 routine brain MRI scans, achieving AUCs of 91.6%, 89.6%, 85.9%, and 81.5% for molecular marker prediction and progression assessment across multicenter datasets. Gomaa et al. (2025) [74] developed a self-supervised multimodal transformer for distinguishing pseudoprogression from true progression in glioblastoma, achieving an external AUC of 75.3%.

Several reviews have consolidated the current state of foundation models in oncologic imaging. Paschali et al. (2025) [62] reviewed foundation models in radiology and identified the absence of rigorous cross-institutional generalization testing as a defining gap in the evidence base. van Veldhuizen et al. (2025) [63] covered opportunities and open challenges related to domain shift, data heterogeneity, and clinical validation across medical imaging modalities. D’Antonoli et al. (2025) [75] reviewed foundation model applications in radiology with emphasis on interpretability and clinical accountability, and Fang et al. (2025) [64] surveyed the broader transition from task-specific architectures to general-purpose pretrained systems across medical imaging.

Despite the genuine progress these models represent, several limitations deserve attention. SSL reduces dependence on labeled data but not on data distribution. Foundation models pretrained predominantly at high-volume academic centers reflect the acquisition protocols and patient demographics of those specific contexts [76,77], and when deployed elsewhere, pretrained representations may transfer pretraining biases as reliably as biological signal [78]. The SSL objective itself adds further constraints: contrastive methods assume certain augmentations are semantically neutral, which is harder to defend in CT or MRI than in natural images [79,80], while masked autoencoding tends to encode local texture statistics rather than the global anatomical reasoning that oncologic predictions often require [81]. Neither objective was designed with clinical semantics in mind, and this misalignment does not disappear through fine-tuning alone. Interpretability is also harder at this scale. Foundation model representations are distributed across high-dimensional latent spaces that resist direct interrogation [75,82]. Probing techniques can reveal what biological information is encoded [62], but they cannot show how that information drives predictions or whether decision-relevant features reflect biology or pretraining artifacts [83,84].

A further caveat that deserves explicit discussion is the gap between benchmark performance and clinical utility. Foundation models perform impressively on curated, retrospective datasets, often drawn from high-volume academic centers. That alone does not show they transfer across organs, modalities, centers, populations, or clinical workflows. These models are typically evaluated under controlled conditions—standardized acquisition protocols, curated patient cohorts, and well-defined endpoints—and such conditions rarely match the messiness of everyday clinical practice. There is also a bias problem rooted in the pretraining data itself. Foundation models are trained mostly on data from academic medical centers in high-income countries. That data may not reflect the demographic diversity, disease spectrum, or imaging protocols found elsewhere. The result can be systematic underperformance in underserved populations or in regions with different healthcare infrastructure, which raises real concerns about fairness and equity once these models are deployed. Heavy reliance on high-volume academic centers raises questions about reproducibility and generalizability. A model that performs well on data from one institution can fail on data from another, simply because scanners differ, acquisition parameters differ, patient populations differ, and clinical workflows differ. For this reason, rigorous external validation across diverse, multi-institutional cohorts is necessary before anyone claims clinical readiness, and ongoing post-deployment monitoring matters too, to catch performance drift and bias once a model is actually in use.

Foundation models are the most capable tools oncologic imaging has seen. The data efficiency gains are real, benchmark performance is strong, and cross-task transferability is something no earlier approach could offer. The central question now is not whether these models perform well under controlled conditions—they do—but whether that performance holds when they meet the full variability of real clinical environments. That is the challenge the field has yet to fully answer—and it is precisely what the following sections examine: how acquisition variability, validation deficiencies, and interpretability gaps continue to block even the most capable models from reaching the clinic. Table 4 summarizes representative studies using foundation models and self-supervised learning in oncologic imaging.

## 4. The Translational Gap in Clinical Settings

The “Translational Gap” refers to the ongoing challenge in extending predictive models from laboratory outcomes to real-world clinical support systems. Concerning oncology, it depends on a number of factors that will be introduced and discussed in the following subsections.

### 4.1. Acquisition Variability

Acquisition variability describes the variability in the image data that is due solely to the way in which the images were acquired. It means that if the same patient, with the same tumor, were scanned on two different machines or in slightly different ways, the two images would appear numerically different. While a human radiologist can visually interpret both of these images, AI systems and radiomics (which use hundreds of data points extracted from an image) can be very sensitive to these numerical differences, and this can make it difficult to identify the true signal of the tumor.

To understand and mitigate acquisition variability, it is useful to distinguish between its different sources, as each affects radiomic features in different ways and requires different mitigation strategies. **Scanner variability** refers to differences between imaging devices from different vendors (e.g., GE, Siemens, Philips) or even between different models from the same vendor. These differences arise from variations in hardware design, detector technology, magnetic field homogeneity (in MRI), and system calibration. Scanner variability is a major source of batch effects in multi-center studies and can lead to features that are more reflective of the scanner than of the underlying biology [85]. **Acquisition protocol** encompasses the specific settings used during image acquisition. In CT, these include tube voltage (kVp) and tube current (mAs), which affect image noise and contrast; in MRI, they include pulse sequences, echo time (TE), repetition time (TR), flip angle, and magnetic field strength (1.5 T vs. 3 T); in PET, they include tracer dose, uptake time, and acquisition duration. Variations in these parameters can substantially alter radiomic feature values, particularly texture and first-order statistics [9]. **Reconstruction** refers to the algorithms and parameters used to convert raw data into images. In CT, reconstruction kernel (smooth vs. sharp) and iterative reconstruction strength affect spatial resolution and noise texture; in MRI, reconstruction algorithms and filtering affect image appearance; in PET, reconstruction methods (e.g., OSEM, filtered back-projection) and matrix size affect feature reliability [86]. Studies have shown that radiomic features are often more sensitive to reconstruction parameters than to other acquisition settings [87]. **Contrast administration** affects image intensity and enhancement patterns, particularly in CT and MRI. Variations in contrast injection rate, total volume injected, scan timing relative to injection, and the phase of enhancement (e.g., arterial, portal venous, delayed) can drastically alter tumor enhancement patterns and, consequently, radiomic features. Portal venous phase timing variations of even seconds can alter density measurements in liver metastases [88]. **Segmentation** refers to the delineation of the region of interest (ROI) from which radiomic features are extracted. Variability in segmentation arises from differences in manual contouring between readers (inter-observer variability), differences in the same reader over time (intra-observer variability), and differences between manual, semi-automated, and automated segmentation methods. Even small differences in ROI boundaries can substantially affect shape and texture features [24,25]. **Preprocessing** encompasses the steps applied to images before feature extraction, including resampling to isotropic voxels, intensity normalization, discretization, and filtering. Different preprocessing pipelines can produce substantially different feature values, and the lack of standardized preprocessing protocols is a major barrier to reproducibility in radiomics [22]. For example, the choice of bin width in intensity discretization affects texture feature values, and different normalization methods can lead to different feature distributions. **Patient physiological factors** refer to patient-specific characteristics that affect image appearance independently of the disease. In CT, these include patient body habitus, which affects image noise and attenuation; in MRI, cardiac output and blood flow affect contrast enhancement kinetics; in PET, blood glucose levels affect tracer uptake. Patient positioning, breath-hold depth, and the angle of the patient’s arms can also influence organ orientation and lesion appearance. These factors are particularly challenging because they are difficult to standardize across patients and institutions. Each imaging modality has its own specific sources of variability. In **CT**, the primary sources are scanner manufacturer, tube voltage, tube current, reconstruction kernel, slice thickness, and contrast phase. In **MRI**, magnetic field strength, pulse sequence type, TE, TR, flip angle, and the use of contrast agents are major sources of variability. In **PET**, tracer type, injected dose, uptake time, reconstruction algorithm, and matrix size are key sources. In **ultrasound**, operator dependence, transducer frequency, gain settings, and depth affect image quality and feature stability. In **digital pathology**, scanner type, magnification, stain quality, and slide preparation affect image properties and feature reproducibility. Recognizing these modality-specific sources of variability is essential for designing robust radiomics studies and for interpreting results across different imaging platforms and clinical settings. Beyond these specific sources, acquisition-related variation can be broadly categorized into random effects and systematic biases [10]. Random effects are stochastic variations (e.g., day-to-day variations in scanner performance) that are challenging to control but can be quantified through methods such as test–retest imaging and perturbation analysis [24,25]. Systematic biases arise from differences in scanner hardware, acquisition protocols, and reconstruction settings. Emerging sensor technologies are also introducing new sources of variability while simultaneously offering opportunities for more standardized data collection. Photon-counting detectors, for example, provide energy-resolved data that can improve material decomposition and reduce beam-hardening artifacts, but they also introduce new reconstruction parameters that affect radiomic feature stability. Similarly, simultaneous PET/MRI systems integrate multiple sensor types within a single acquisition, enabling co-registered functional and anatomical data but introducing new challenges related to sensor cross-talk and sequence-dependent signal variations. In ultrasound, the transition from conventional piezoelectric transducers to capacitive micromachined ultrasonic transducers (CMUTs) and single-crystal technologies is affecting bandwidth and image quality characteristics that influence texture-based radiomic features. Understanding these sensor-level characteristics is essential for designing robust radiomics studies and for interpreting the performance of machine learning models across different imaging platforms.

Finally, some biases depend on the patient. For example, the angle of the patient’s arms, the depth of their breath-hold, or even their position on the table may influence the orientation of organs and lesions. The physiological state is another source of bias. For instance, blood glucose levels may impact PET images, or cardiac output may influence contrast agent uptake. Understanding these sources of variability is essential not only for designing robust radiomics studies but also for interpreting the performance of machine learning models in clinical settings—a theme we return to in the following sections.

### 4.2. Dataset Homogeneity and “Small Data” Challenges

From a machine learning perspective, the main challenge in oncologic radiology today is not acquiring images but building datasets that satisfy the conditions for statistical well-posedness, i.e., structured in such a way that the learning problem is solvable and generalizable. While healthcare systems have a huge amount of images, the available sample size is often significantly smaller than the number of modern model parameters, and where the risk of overfitting is a major concern [42]. A related concern is dataset shift, by which patterns that are not actually related to the disease are introduced during the dataset curation phase, and this can be particularly problematic in small data settings [89]. The small-data problem in oncology is structural since the number of available, labeled cases is inherently low. This is exacerbated by the cost of obtaining high-quality labels, which often require histopathological confirmation or pixel-level annotations from expert radiologists. This way, a model is never exposed to the full heterogeneity of the target domain during training, and then it learns a decision boundary that is highly specific to that narrow support, leading to a failure of domain adaptation [90]. However, the “small data” problem is not monolithic: several distinct but interrelated factors affect model validity in different ways. **Small sample size** refers to the limited number of patients or images available for training. This structural problem in oncology restricts statistical power and increases the risk of overfitting, particularly when combined with high-dimensional feature spaces. Even when a model achieves high performance on a small training set, this often reflects memorization of dataset-specific patterns rather than learning generalizable biological features, leading to poor performance on new, independent cohorts. **High dimensionality** refers to the large number of features extracted from each sample—whether hand-crafted radiomics features (often hundreds or thousands) or deep learning-derived latent representations (potentially millions of parameters). When the number of features approaches or exceeds the number of samples, the model can easily fit noise rather than signal, a phenomenon known as the curse of dimensionality. **Limited population diversity** refers to the lack of representation of different patient subgroups, demographics, disease stages, or imaging protocols in the training data. Even with an adequate sample size and proper validation, a model trained on a homogeneous population may not generalize to diverse clinical settings. This is particularly problematic in oncology, where patient populations vary significantly across institutions, geographic regions, and healthcare systems. Moreover, when considering small sample sizes in combination with large-dimensional feature spaces, whether based on hand-crafted radiomics or deep learning-based latent space representations, there exists a phenomenon known as the curse of dimensionality. When operating in a large-dimensional space, all points become equivalent in terms of sparsity, which in turn renders distance metrics ineffective. Moreover, there exists a greater likelihood of false correlations [27]. Without explicit regularization, dimensionality reduction techniques such as autoencoders or Principal Component Analysis (PCA), or even strong inductive biases such as CNN architectures that inherently promote spatial understanding, there exists a risk of finding patterns that, although statistically significant on a small sample size, may not generalize well [91].

### 4.3. Deficiencies in External Validation Protocols

A critical challenge in translating radiomics models to clinical practice is the gap between model performance in controlled research settings and performance in real-world clinical environments. However, the literature often uses terms such as external validation, multi-institutional validation, prospective validation, transportability, generalization, and post-deployment monitoring interchangeably, obscuring important distinctions. To provide conceptual clarity, we define these terms as follows: **External validation** refers to the evaluation of a model’s performance on data from an independent institution or cohort distinct from the training set, assessing whether the model can generalize beyond the development dataset. **Multi-institutional validation** is a specific form of external validation where the test data come from multiple institutions, providing evidence of robustness across different clinical settings and acquisition protocols. **Prospective validation** involves evaluating a model in real-time clinical workflows, with data collected prospectively (i.e., after model development), representing the highest level of evidence for clinical utility. **Transportability** refers to the ability of a model to generalize across similar clinical settings (e.g., different hospitals within the same healthcare system or country), typically assessed through validation on data from comparable institutions. **Generalization under distribution shift** refers to the ability of a model to maintain performance when the test data distribution differs from the training distribution (e.g., different scanner vendors, acquisition protocols, patient demographics, or disease prevalence). This represents a stronger test of model robustness than transportability. **Post-deployment monitoring** refers to the ongoing surveillance of model performance after clinical implementation, including detection of performance drift due to changes in patient populations, imaging protocols, equipment upgrades, or software updates. This is a regulatory and clinical imperative for maintaining trust and safety. Throughout this section, we use these terms consistently to distinguish between different levels of validation rigor and to emphasize that internal validation, while necessary, is insufficient for establishing clinical readiness.

Beyond the general limitations of validation protocols, specific methodological issues contribute to overly optimistic performance estimates in the radiomics literature. These include overfitting in small-sample studies—particularly those reporting near-perfect accuracy (>95–99%)—data leakage due to inadequate patient-level splitting (e.g., using multiple images from the same patient across training and test sets), feature selection bias when feature selection is performed on the entire dataset before cross-validation, and the reuse of public benchmark datasets (e.g., BraTS, TCGA, Figshare, Kaggle) without proper cross-dataset generalization testing. Studies reporting exceptionally high performance on small, single-center, or public datasets should therefore be interpreted with caution, as such results rarely replicate in independent, larger cohorts. The substantial performance drop from internal to external validation further underscores the limited generalizability of models developed under these conditions.

Even with an adequate training set, the path to clinical utility is blocked by inadequate methodologies for model validation. This has resulted in a “generalizability gap,” where there is a systematic overestimation of model performance in the real world based on overly optimistic internal performance metrics [92].

A recent systematic review on the performance of AI-based diagnostic models for radiology articles between 2022 and 2025 reported that the AUC for internal validation ranged from 0.76 to 0.95. However, the AUC dropped by 0.03 for the median for the external validation. In addition, the specificity dropped by 24 percentage points [92].

In a study analyzing 86 deep learning algorithms for radiology, 81% showed a drop in the performance of the algorithm on external validation. In addition, a quarter of the algorithms showed a drop in performance by 0.10 points or more [93]. This is not a matter of statistical fluctuations but rather a failure to generalize the model.

The fundamental root of this issue is the blurring of lines between internal validation and external validation within the context of model development. Indeed, several research papers on deep learning include internal cross-validation and any type of external validation [94].

However, even in the case of external validation, it is often marred by design flaws, with the most common one being the use of so-called “external” validation sets, which are actually from the same institution, patient population, or acquisition protocol as the training set, thereby merely duplicating internal validation with a different name [95]. In fact, for external validation to be meaningful, a distribution shift must be present, which includes differences in manufacturers, protocols, patient populations, or disease prevalence, which actually tests whether the model has learned invariant features or merely dataset-specific artifacts [96].

Inadequate validation can result in academic overclaiming and can also lead to automating and amplifying biases. Previous research has demonstrated that radiology-based AI models can unintentionally learn demographic “shortcuts” from medical images, with models that are less reliant on demographic attributes being more globally optimal, i.e., retaining higher accuracy and fairness on a wide range of test populations [92]. Inadequate validation can mean that these biases are not apparent until after deployment, where they become a patient safety risk.

More recent research has set out to develop more stringent validation methodologies. Indeed, both the Developmental and Exploratory Clinical Investigations of DEcision support systems driven by Artificial Intelligence (DECIDE-AI) and Transparent Reporting of a multivariable prediction model for Individual Prognosis or Diagnosis AI (TRIPOD-AI) reporting checklists now specifically address the need for transparent validation and testing of AI systems [92]. Moreover, the European Society of Radiology has developed a set of surveillance standards that require monitoring of AI system performance drift, threshold-based alerts with action plans, and systematic logging of edge-case failures to detect performance gaps for subgroups of patients [97]. This acknowledges that validation is an ongoing process, not a one-time event.

From the methodological point of view, the way forward involves several specific steps: the need for external validation datasets to be independent, collected at different institutions, and preferably different countries or healthcare systems with documented differences in acquisition parameters and case mix to assess generalization under distribution shift; the necessity of including explicit subgroup analyses to detect performance disparities by age, sex, race, and disease subtypes; the importance of reporting calibration, i.e., the relationship between the predicted probabilities and the observed outcomes, along with the performance of the model in terms of discrimination, as the lack of calibration may mislead clinical decisions even if the ranking accuracy of the model is maintained [96]; and finally, the need to move forward with prospective validation rather than retrospective validation, as the latter, even on external data, cannot fully capture the complexities of integration into clinical workflows [98].

This problem is further exacerbated by potentially reductionist single-metric comparisons. Traditionally, a plethora of different metrics are reported in lengthy tables. While certain models may demonstrate high performance on some of these metrics and underperform on others, it becomes challenging to establish which particular model would prove better-suited for deployment in a clinical setting. This may prevent the choice of the best-performing model due to the need to balance sensitivity and specificity, among other important criteria. A step towards overcoming this limitation could be taken by introducing a comprehensive evaluation technique that would aggregate a number of criteria and produce an easily interpretable evaluation result. One such approach is called the Polygon Area Metric (PAM) [99]. It implies calculating the area of a polygon constructed using the values of various metrics, such as sensitivity, specificity, AUC, and F1-score. As such, the area of the resulting polygon serves as an indicator of better model performance. By synthesizing multiple performance dimensions into a single score, PAM could serve as a valuable tool for standardizing model evaluation, thereby helping to bridge the gap between promising research prototypes and clinically deployable systems.

It is important to note that retrospective external validation, while valuable for assessing transportability and generalization under distribution shift, cannot fully capture the complexities of prospective clinical integration, including workflow integration, human–AI interaction, and real-time decision-making. Furthermore, post-deployment monitoring is essential to detect performance drift and ensure that models remain safe and effective over time. The emerging consensus is that internal validation is necessary but utterly insufficient to establish trust in clinical performance and that any model that has not been subject to rigorous, independent, and multi-institutional validation should be considered research prototypes, not clinical tools. The field must move from a culture of celebrating internal performance metrics to one that demands evidence of transportability and generalization under distribution shift as a precondition for publication, regulatory clearance, and clinical adoption.

### 4.4. Black-Box Nature and Lack of Uncertainty Reporting

While the performance of the model on an external validation set may be robust, a key challenge to the clinical adoption of the model remains: the “black box” challenge, where the lack of transparency in the decision-making process of the model and the inability to effectively communicate uncertainty through the decision-making process of the model create a challenge for the trust required for high-stakes oncology decision-making, as the clinician cannot readily evaluate whether the decision should be trusted or challenged [100,101].

The essence of the problem lies in the architecture of the models themselves. Modern deep neural networks, especially convolutional neural networks (CNNs) and vision transformers, are complex non-linear function approximators with millions of parameters. The representations learned by these models are distributed and hierarchical, making it difficult to understand their workings [101]. Even though these models have high predictive capabilities, they fail to provide any explanation for their output for a given input. This is a problem that is untenable in a clinical scenario where life-or-death decisions are being made.

### 4.5. Human–AI Workflow Integration Barriers

Even when models demonstrate robust performance, interpretability, and uncertainty calibration, a final barrier stands between algorithmic promise and clinical impact: seamless integration into real-world radiology workflows. This “implementation chasm” reflects the fundamental disconnect between model-centric development and the socio-technical realities of clinical practice [102]. The question is no longer merely whether an AI model can perform a task but whether it should be integrated, how it will be used, and who bears responsibility when human–AI collaboration fails [103].

The prevailing evaluation paradigm remains model-centric: algorithms are assessed on standalone metrics. Yet clinical practice is inherently team-based, dynamic, and context-dependent [102]. Implementation failures often stem not from algorithmic inadequacy but from misalignment with existing workflows and the absence of specialized expertise to bridge technical development and clinical reality.

Clinicians do not respond homogeneously to AI assistance. A study of response-adaptive radiotherapy found that at the population level, AI assistance produced no statistically significant shifts, yet at the individual level, approximately half of all cases resulted in decision adjustments correlated with dissimilarity between clinician and AI recommendations [104]. Decisions were influenced by prior knowledge, patient state, model transparency, and perceived biases—underscoring that human–AI collaboration cannot be reduced to simple performance narratives.

Central to successful integration is trust calibration—aligning clinician trust with actual AI capability. Miscalibration manifests as overtrust and undertrust [105]. Machines with learned self-assessment improve human trust by approximately 40% and team performance by 5%, demonstrating that how machines communicate uncertainty matters as much as what they predict.

Integration carries cognitive and professional consequences [106]. Automation bias can lead clinicians to accept incorrect recommendations without verification. Algorithmic aversion leads to underutilization of valuable support. Deskilling represents a longer-term risk: as AI assumes tasks like lesion detection, trainees may miss opportunities for deliberate practice [107]. Paradoxically, AI tools intended to reduce workload may initially increase it, as clinicians must review and document AI outputs [108].

In radiology, the prevailing physician-in-the-loop model maintains diagnostic liability with the radiologist while leveraging AI for specific sub-tasks [106]. Responsible implementation requires formal governance: continuous post-market surveillance, clear liability frameworks, validated explainability, and systematic AI literacy training [97].

## 5. Discussion

Radiomics research involves an extraordinarily wide range of methodological decisions at every stage—from defining the study objective and preprocessing images to extracting features and conducting statistical modeling and validation. While this flexibility encourages innovation, it has also led to a rise in studies that prioritize superficial novelty over substantive scientific advancement [109]. Superficial novelty typically involves introducing a new or only slightly modified study aim that implies a clear endpoint for clinical utility—such as applying radiomics to a different disease, adopting an updated classification system, or making minor adjustments to existing pipelines. Although such studies may seem innovative, they seldom deepen our understanding of radiomics’ limitations or underlying dependencies. For instance, proposing a novel feature extraction technique—like a deep learning algorithm with modest architectural tweaks—might produce improved accuracy on the surface. However, without rigorous evaluation of fundamental assumptions (e.g., feature stability across varying conditions), both reproducibility and clinical relevance remain uncertain, even if the approach appears methodologically novel. To fill this gap, Radiomics Quality Score (RQS) 2.0 is an updated tool designed to assess the scientific rigor and quality of radiomics studies [110]. By providing a comprehensive scoring system and a clear roadmap for development (RRLs), RQS 2.0 helps researchers and reviewers benchmark their work, identify gaps, and focus on the steps necessary to create robust, reliable, and clinically useful tools for precision oncology.

Going into the challenging aspects of how to address the translational gap, in the traditional machine learning era, acquisition variability and small data were addressed through separate, largely retrospective strategies. For acquisition variability, the dominant approach was post hoc harmonization of hand-crafted radiomics features. The ComBat method, originally developed for genomic data, was widely adopted to remove batch effects by modeling and subtracting scanner-specific biases while preserving biological signal [23]. Image preprocessing—including intensity normalization, resampling to isotropic voxels, and standardized discretization—became standard practice to reduce variability before feature extraction. Test–retest imaging and perturbation analysis were used to identify highly repeatable features, with only robust features retained for modeling [24,25].

For the small-data problem, traditional approaches focused on feature engineering and regularization. While these approaches improved reproducibility, they remained fundamentally limited. Modern Machine Learning has shifted the paradigm from retrospective correction to prospective invariance learning, addressing acquisition variability and small data within a unified framework. The core insight is that both challenges can be mitigated by learning representations that are simultaneously robust to acquisition variations and data-efficient—capturing maximal information from limited labels by leveraging vast quantities of unlabeled data.

Concerning explainability, the machine learning community has recently responded with a suite of explainable AI (XAI) techniques designed to render model decisions interpretable. In medical imaging, saliency-based methods—particularly Gradient-Weighted Class Activation Mapping (Grad-CAM) and its variants—have become the de facto standard for generating visual explanations [100]. These methods produce heatmaps highlighting regions of the input image that contributed most to the model’s decision. Similarly, Shapley Additive Explanations (SHAP) and Local Interpretable Model-Agnostic Explanations (LIME) have been adapted to provide feature-level attributions.

However, a growing body of evidence reveals that these explanations are often unfaithful or unstable. A systematic review of XAI in cardiovascular imaging found that most studies rely on qualitative assessment of saliency maps, with little to no validation of whether the highlighted regions actually correspond to clinically meaningful features [100]. The review identified that Grad-CAM was the most frequently employed method, yet its outputs were rarely subjected to quantitative faithfulness tests such as deletion or insertion experiments, which measure whether removing highlighted regions actually degrades model performance as expected.

This lack of validation has profound implications. Studies have demonstrated that popular post hoc explanations can be highly sensitive to minor perturbations in input data or model parameters, producing dramatically different attribution maps for nearly identical inputs [91]. More concerningly, explanations can appear plausible—highlighting anatomical structures that “make sense” to human observers—while being entirely disconnected from the model’s actual decision logic. This phenomenon, termed “explanation artifact,” creates a dangerous illusion of understanding: clinicians believe they comprehend the model’s reasoning when in fact they are being misled by visually appealing but unfaithful heatmaps [111].

Recent work in head-and-neck cancer radiotherapy segmentation has begun to address this challenge through rigorous quantitative evaluation. Strijbis and colleagues compared seven XAI methods for parotid gland segmentation, assessing not only visual plausibility but also the correlation between attribution patterns and segmentation quality [111]. They found that PatternNet and guided backpropagation produced attributions that aligned most closely with expert reasoning, focusing on soft-tissue boundaries rather than high-density bone artifacts. Importantly, they demonstrated that topological analysis of attribution maps—using persistent homology to characterize Euler characteristic profiles—could predict segmentation failures a priori, with specific attribution patterns achieving sensitivity above 0.85 and specificity above 0.90 for identifying insufficient contours. This work represents a critical shift: from using XAI merely for post hoc visualization to leveraging it as a prospective quality assurance tool.

The emerging consensus, articulated in recent guideline proposals, is that explainability claims must be accompanied by rigorous validation. A minimum evidence package for XAI should include: (1) sanity checks to ensure explanations are sensitive to model parameters and data; (2) quantitative faithfulness tests (deletion/insertion, Remove and Retrain (ROAR), Iterative Removal of Features (IROF)); (3) stability analyses across similar inputs; (4) concept-level validation (e.g., Testing with Concept Activation Vectors (TCAV) with statistical testing); and (5) prospective human factors studies demonstrating that explanations improve clinical decisions without inducing automation bias [91]. Without such validation, it may result in heatmaps being used as illustrations and not mechanism explanations.

Parallel to the explainability gap is a profound deficiency in Uncertainty Quantification (UQ). Standard deep learning models produce point estimates—a single prediction with no indication of confidence. In oncology, where treatment decisions hinge on probabilistic assessments (e.g., “what is the likelihood this nodule is malignant?”), the absence of well-calibrated uncertainty estimates is a critical limitation. A comprehensive review of UQ techniques in radiotherapy [112] identified that while segmentation is the most common task addressed by UQ methods, the integration of uncertainty estimates into clinical workflows remains nascent. The review distinguishes between two fundamental types of uncertainty: aleatoric uncertainty, arising from inherent noise in the data (e.g., image acquisition artifacts, inter-observer variability in contouring), and epistemic uncertainty, arising from model limitations (e.g., insufficient training data, distribution shift). Aleatoric uncertainty is irreducible but can be characterized; epistemic uncertainty is reducible through better data and model design. The technical landscape for UQ has matured considerably. Bayesian neural networks provide a principled foundation for probabilistic inference but remain computationally prohibitive for large-scale deployment. Monte Carlo dropout, which approximates Bayesian inference by enabling dropout at test time, has emerged as a popular lightweight alternative. Ensemble methods—training multiple models and examining prediction variance—offer another robust approach, though at increased computational cost. More recent innovations include evidential deep learning, which places prior distributions over model parameters to enable single-pass uncertainty estimation [113].

Despite these methodological advances, adoption in clinical studies lags. The majority of published models report only point estimate metrics (accuracy, AUC, Dice) without any uncertainty characterization. This omission is not merely academic: a miscalibrated model can produce predictions that are confidently wrong, with potentially catastrophic consequences. For example, a model estimating tumor volume might provide a point estimate of 15.2 cm^3^ when the true volume could plausibly range from 8 to 22 cm^3^, depending on segmentation ambiguity. Without confidence intervals, the clinician cannot distinguish between high-certainty and low-certainty predictions, undermining informed decision-making.

Recent work has begun to address this through conformal prediction, a distribution-free framework that provides prediction sets with guaranteed coverage under minimal assumptions [114,115]. Cheung and colleagues introduced COMPASS (Conformal Metric Perturbation Along Sensitive Subspaces), which generates efficient, metric-based prediction intervals by perturbing intermediate features along directions maximally sensitive to the target metric [114]. Applied to segmentation tasks, including skin lesion and thyroid nodule analysis, COMPASS produced significantly tighter intervals than traditional conformal baselines while maintaining valid coverage. Similarly, Maes and colleagues proposed a confidence-aware framework for ratio-based biomarkers (e.g., necrotic tissue proportion), incorporating a post hoc calibration module that can be applied using internal hospital data without retraining [115]. Their method enables tunable confidence levels, allowing adaptation to clinical practice requirements.

To operationalize uncertainty quantification for clinical deployment, we recommend that studies report the following metrics and procedures as minimum standards: (i) Calibration curves (reliability diagrams) plotting observed frequency against predicted probability across deciles, with visual assessment of calibration slope and intercept; (ii) Brier score as a proper scoring rule measuring overall probabilistic accuracy (mean squared error between predicted probabilities and binary outcomes), with lower values indicating better calibration; (iii) Expected Calibration Error (ECE) as a summary measure of miscalibration, computed as the weighted average of the absolute difference between predicted probabilities and observed frequencies across bins; (iv) 95% confidence intervals for all primary performance metrics (AUC, accuracy, Dice, C-index), estimated via bootstrapping with ≥1000 resamples; (v) Subgroup-specific calibration metrics reported separately for internal and external validation cohorts and for clinically relevant subgroups (by scanner type, institution, age, sex, disease stage) to detect calibration decay under distribution shift; and (vi) Conformal prediction (e.g., COMPASS) for generating distribution-free prediction sets with guaranteed coverage, reporting both coverage probability and average interval width.

The importance of calibration extends beyond individual predictions to subgroup performance. Models may be well-calibrated overall yet systematically miscalibrated for specific patient subpopulations defined by age, sex, race, or disease subtype. Detection of such calibration failures requires explicit subgroup analyses, which remain rare in the literature [116]. Without them, models risk perpetuating or amplifying healthcare disparities under the guise of algorithmic objectivity.

Paths Forward: Toward Trustworthy AI Addressing the black-box and uncertainty deficits requires a multi-level strategy that spans technical innovation, validation rigor, and clinical integration.

At the technical level, the field must move beyond post hoc explanations toward architectures with intrinsic interpretability. Attention mechanisms, particularly in transformer architectures, offer some transparency by design, as attention weights can be inspected to understand which input regions the model focuses on. Concept bottleneck models, which first predict clinically meaningful concepts (e.g., “presence of spiculation,” “margin irregularity”) before making final predictions, provide another promising direction: they force reasoning through interpretable intermediate representations that clinicians can verify.

For UQ, the path forward involves broader adoption of ensemble methods and conformal prediction as standard practice. Rather than treating uncertainty quantification as an optional add-on, it should be considered a core component of any model intended for clinical deployment. This shift requires corresponding changes in reporting standards: journals and conferences should mandate uncertainty reporting alongside traditional performance metrics.

At the validation level, human factors studies are essential. Even perfect explanations are useless if clinicians cannot effectively integrate them into decision-making. Studies must assess whether XAI tools actually improve diagnostic accuracy, reduce cognitive load, and increase appropriate trust—or whether they induce automation bias and over-reliance. Early evidence suggests that well-designed explanations can enhance clinician performance, but the effect is highly dependent on presentation format and user training [101].

At the regulatory level, frameworks such as the EU’s proposed AI Act and the U.S. Food and Drug Administration’s (FDA) updated guidance on Software as a Medical Device (SaMD) are beginning to mandate transparency and uncertainty communication. The European Society of Radiology has issued guidelines calling for “validated explainability coupled with uncertainty-aware selective workflows,” recognizing that trustworthy AI must know when to defer to human judgment [97]. These regulatory developments will likely accelerate the adoption of rigorous XAI and UQ practices.

Ultimately, the transition from black-box models to trustworthy clinical tools requires a fundamental cultural shift: from prioritizing raw accuracy to valuing reliability, interpretability, and calibrated communication of confidence. Models that cannot explain themselves or quantify their own uncertainty have no place in high-stakes oncology. The field must hold itself to a higher standard, recognizing that trust is not granted by technical performance alone but earned through demonstrated transparency and reliability at every level of the clinical workflow.

The deficiencies of validation protocols were initially addressed through structured reporting guidelines that explicitly mandated external validation. TRIPOD-AI and DECIDE-AI required authors to clearly distinguish between development and validation phases, report performance separately, and describe validation population characteristics [92]. These guidelines established that external validation was not optional but essential. Concurrently, quality assessment tools emerged to systematically evaluate validation rigor. The Must AI Criteria-10 (MAIC-10) checklist provided a structured framework for assessing study quality across domains, including validation methodology [94]. Application of MAIC-10 revealed mean quality scores around 5.6 out of 10, with validation items frequently incomplete—providing both a diagnosis and a roadmap for improvement. The field developed a more sophisticated understanding of what constitutes true external validation. A critical distinction emerged between transportability (generalizing across similar institutions) and generalizability (performing across fundamentally different conditions). True external validation requires a distribution shift—differences in scanner manufacturers, protocols, patient demographics, or healthcare systems [95,96]. Only when performance is assessed under such a shift can claims of generalizability be substantiated. Modern validation studies now explicitly document these differences. External validation datasets must differ along clinically relevant dimensions, and performance must be reported separately for each distinct population and acquisition context [96]. This represents a shift from validation as a checkbox to validation as a scientific inquiry into model boundaries. A parallel advance has been the recognition that external validation must extend to subgroup performance. Models may be well-calibrated overall yet systematically biased for specific patient subpopulations defined by age, sex, race, or disease subtype [116]. Studies have shown that radiology AI models can inadvertently learn demographic “shortcuts,” and models with less demographic dependence tend to be more globally optimal [92]. Modern validation frameworks now mandate disaggregated performance reporting to detect and mitigate such disparities before deployment.

The most significant evolution has been the shift from retrospective to prospective validation and from one-time assessment to continuous monitoring. Retrospective validation, even on external data, cannot capture the complexities of clinical workflow integration—time pressure, human–AI interaction, and real-world case mix [117]. Prospective studies enroll patients in real time, with AI predictions generated during clinical workflow and compared against actual outcomes, providing the only true test of clinical utility.

Even prospective validation at a single time point is insufficient. Post-market surveillance has emerged as a regulatory and clinical imperative [97]. AI models must be monitored for performance drift due to changes in patient populations, imaging protocols, equipment upgrades, or software updates. The European Society of Radiology now mandates comprehensive post-market monitoring, including routine performance drift detection and systematic logging of edge-case failures [97].

Enabling continuous validation requires technical infrastructure absent in the traditional era. Federated learning platforms have been repurposed for distributed validation: models can be evaluated at multiple institutions without moving patient data [118]. Automated performance monitoring systems integrated into clinical PACS (Picture Archiving and Communication System) can track model performance against ground truth as it becomes available. Standardized data formats and interoperable APIs enable validation across different healthcare systems without custom integration.

Regulatory frameworks have accelerated the adoption of rigorous validation. FDA guidance on Software as a Medical Device requires evidence of clinical validation across intended use populations. The EU Medical Device Regulation and proposed AI Act mandate conformity assessments, including real-world validation. The National Institute for Health and Care Excellence (NICE) has established evidence standards explicitly requiring external validation [119]. These developments have transformed validation from a scientific nicety to a legal requirement.

Validation is no longer viewed as a final step before publication but as a continuous scientific discipline spanning the entire model lifecycle. The new paradigm rests on prospective design, distribution shift, subgroup analysis, continuous monitoring, and regulatory oversight. Models that have not undergone such rigorous validation are recognized as research prototypes, not deployable clinical tools. The field has moved from celebrating internal performance metrics to demanding evidence of transportability as a precondition for clinical adoption.

Moving beyond mere integration requires reimagining human–AI collaboration as a genuinely synergistic partnership rather than a tool-using relationship. This vision demands several paradigm shifts.

First, adaptive collaboration frameworks must replace static decision-support models. Future systems will dynamically modulate their autonomy based on real-time assessment of both task complexity and clinician cognitive state. When a radiologist is fatigued or the case is ambiguous, the AI might offer more detailed explanations; when the clinician is fully engaged and the case is routine, the AI might recede into the background. This requires models that not only predict but also sense their collaborative context.

Second, bidirectional learning must become the norm. Current workflows treat AI as a fixed model and clinicians as static recipients of its outputs. In reality, both should learn from each other. AI systems should capture clinician corrections and refinements as training signals for continuous improvement. Clinicians should receive feedback on their own performance relative to AI suggestions, supporting metacognition and skill development rather than deskilling.

Third, shared cognitive work requires new interface paradigms. Rather than presenting predictions as discrete outputs, future systems might visualize decision spaces, highlight regions of uncertainty, and support interactive exploration of alternative interpretations. The goal is not to replace clinical reasoning but to augment it—providing information in forms that integrate with rather than interrupt natural cognitive workflows.

Fourth, responsibility frameworks must evolve from liability allocation to shared accountability. The physician-in-the-loop model maintains ultimate responsibility with the clinician, but this is a legal necessity rather than an optimal collaborative structure. Future frameworks might recognize distributed cognition as the reality of clinical practice, with shared responsibility among developers, institutions, and clinicians supported by clear standards for each party’s contributions.

Finally, training and education must transform. Radiology residents need not only AI literacy but also experience in collaborative human–AI reasoning. Curricula should include simulated AI-assisted interpretations, exercises in appropriate trust calibration, and explicit instruction in when and how to override algorithmic recommendations. The radiologist of the future will not just be an image interpreter but a human–AI collaboration specialist.

The transition from integrated tools to synergistic partners requires moving beyond technical metrics to fundamentally human-centered design. Trust is not earned through performance alone but through demonstrated reliability, transparency, and alignment with clinical workflows and values. The ultimate goal is not AI that performs well in isolation, but AI that makes human clinicians better—enhancing their capabilities, supporting their judgment, and ultimately improving patient outcomes through collaboration that exceeds what either could achieve alone.

## 6. Future Directions for Clinical Robustness

Looking ahead, future research on deep learning for oncologic imaging across X-ray, CT, MRI, ultrasound and PET/CT is expected to be driven by a shift from narrowly supervised, modality-specific models toward large, self-supervised foundation models that can jointly exploit heterogeneous imaging and non-imaging data. These models, typically trained with contrastive or masked objectives on vast collections of unlabeled scans, have already demonstrated improved efficiency and robustness for cancer imaging biomarkers and are poised to become central tools for discovering prognostic signatures when labeled cohorts are limited. A key direction is the development of oncology-specific foundation models that integrate radiographic data across scales and modalities (X-ray, CT, MRI, ultrasound, PET) with text reports, genomics, liquid biopsy and other clinical variables, enabling patient-level risk stratification, treatment recommendation and trial matching beyond single-task classification [63,66,75,82,120,121].

Scaling self-supervised learning will be crucial to this evolution, as it offers a principled strategy to leverage the abundance of unlabeled oncologic imaging while mitigating the scarcity of high-quality annotations. Recent benchmarks in pathology and radiology indicate that in-domain SSL pretraining yields representations that transfer more effectively than ImageNet-based supervision, particularly for downstream survival prediction and radiogenomic tasks, but also highlight open questions regarding objective choice, data curation and cross-site generalization. Extending these paradigms to three-dimensional data and multimodal settings—such as PET/CT, multiparametric MRI and CT fused with omics—will require systematic studies of SSL architectures and fusion strategies, including cross-attention and vision-language objectives that align image features with clinical text and molecular profiles [62,63,75,82,120,121,122,123,124,125,126].

In parallel, generative modeling—especially diffusion-based approaches—will increasingly shape oncologic imaging workflows, moving beyond data augmentation to core roles in reconstruction, denoising and anomaly detection. Diffusion models are already being explored for low-dose CT and low-count PET enhancement, accelerated MRI reconstruction and synthesis of rare tumor presentations, where early studies suggest improvements in image quality and preservation of radiomic features compared with conventional methods, albeit with limited clinical validation to date. Future work will need to establish standardized benchmarks and reporting practices, quantify the downstream impact of generative reconstructions on diagnostic and prognostic models, and develop computationally efficient, explainable variants that can be integrated into time-constrained radiology and radiotherapy pipelines [127,128,129,130,131].

Another important trajectory concerns multimodal and longitudinal modeling of disease trajectories, in which deep networks jointly ingest serial imaging from multiple modalities together with clinical and molecular data to characterize tumor evolution and treatment response. Emerging MRI-omics and PET/CT deep-radiomics studies already illustrate the prognostic value of fusing imaging features with liquid biopsy and other high-dimensional biomarkers, but these efforts remain fragmented and largely retrospective. Future oncology-focused models are likely to adopt architectures with explicit temporal components (e.g., recurrent or Transformer-based sequence modeling) and hierarchical fusion mechanisms that can accommodate heterogeneous, irregularly sampled data streams while providing clinically interpretable outputs for multidisciplinary decision-making [66,75,82,120,121,126,132,133,134].

Finally, across all modalities, there is a growing recognition that methodological advances must be matched by progress in robustness, fairness, and workflow integration if deep learning is to have a durable clinical impact. Reviews of foundation models in radiology and broader AI in medical imaging emphasize the need for rigorous multicenter evaluation, calibration across vendors and demographics, and mechanisms for continual monitoring once systems are deployed. Promising research directions include the design of architectures that explicitly link general-purpose foundation models to specialized task-specific heads via cross-task connections, support efficient adaptation to new scanners or institutions, and enable deployment on resource-constrained platforms such as ultrasound probes or interventional guidance systems. Taken together, these lines of work suggest a future landscape in which deep learning for oncologic imaging is characterized not only by higher predictive performance, but also by tightly integrated, multimodal, and trustworthy systems that operate across the continuum of cancer care [2,62,63,75,120,129,131,133,135].

Sensor innovation will continue to shape the trajectory of radiomics and machine learning in oncology. Emerging technologies such as photon-counting CT, ultra-high-field MRI (7T and above), time-of-flight PET with improved timing resolution, and portable/handheld ultrasound devices with integrated artificial intelligence are expanding the boundaries of what can be measured non-invasively. These sensor advancements generate richer and more complex data—energy-resolved spectra, multi-parametric maps, high-temporal-resolution dynamic data—that create both opportunities and challenges for machine learning: more information to exploit, but also more sources of variability to control. Photon-counting CT, for instance, provides spectral information that could enable novel radiomic biomarkers based on material decomposition, while ultra-high-field MRI offers increased signal-to-noise ratio and spatial resolution that may reveal subtle textural patterns not visible at lower field strengths. However, these sensor-specific characteristics introduce new sources of acquisition variability that must be accounted for in radiomic workflows. Future models will need to either explicitly model sensor-specific signatures through harmonization techniques or learn sensor-invariant representations through domain adaptation and domain generalization strategies, ensuring that predictive models remain robust across different sensor technologies and generations.

Beyond task-specific deep learning models, recent work has introduced agentic AI as an emerging paradigm in radiology and oncology, in which autonomous or semi-autonomous agents orchestrate complex imaging workflows rather than performing isolated predictions. These systems can monitor worklists, select and chain appropriate models (for example, a CT nodule detector, MRI tumor segmenter, or PET/CT radiomics pipeline), and adapt subsequent steps based on intermediate findings and patient-specific context, thereby re-prioritizing urgent studies and reducing time-to-diagnosis in high-volume environments. Conceptually, such agents act as an integration and coordination layer above the convolutional, Transformer, and radiomics models described earlier, transforming them into adaptable, end-to-end imaging pipelines [136].

We emphasize that the AI agents and multi-agent systems discussed above remain investigational and should not be interpreted as mature solutions to the current challenges in validation, explainability, or clinical translation. The evidence base for agentic AI in oncologic radiomics is currently limited to preliminary proof-of-concept studies and reasoned extrapolations from other medical domains. Key open questions—including robustness under distribution shift, error cascades in multi-step pipelines, regulatory pathways, integration with existing clinical workflows, and clear responsibility frameworks—remain largely unresolved. Accordingly, while we identify agentic AI as a promising direction for future research, we do not present it as a ready-to-deploy solution. Rather, we emphasize that the foundational challenges discussed throughout this paper—acquisition variability, validation deficiencies, interpretability gaps, and uncertainty quantification—must be addressed before agentic systems can be responsibly deployed in clinical oncology.

A second, complementary application domain is clinical decision support around oncologic imaging. Systematic reviews of AI-based decision support for tumor boards indicate that algorithmic recommendations can achieve high concordance with multidisciplinary team decisions, suggesting that AI systems are capable of approximating expert consensus for many tumor types. Building on this, multi-agent frameworks have been proposed in which specialized agents—for radiology, pathology, medical and radiation oncology, and literature retrieval—independently analyze imaging, clinical, and molecular data before a synthesis agent aggregates their outputs into structured recommendations with explicit consensus measures. In parallel, conversational and messaging-based agents are being developed as front-ends to these capabilities, surfacing imaging-derived risk scores, staging suggestions, or trial matches within communication tools already used by clinicians and assisting with documentation and patient communication while maintaining human oversight [137,138,139,140].

Taken together, AI agents and multi-agent systems are poised to impact oncologic imaging less by replacing radiologists and oncologists than by coordinating heterogeneous models and data streams, automating low-level but cognitively demanding tasks, and embedding deep learning outputs directly into clinical workflows and tumor-board deliberations. At the same time, current commentaries emphasize that these systems remain largely investigational; key challenges include preventing error cascades in multi-step pipelines, ensuring robustness and calibration across institutions, defining clear responsibility and oversight structures, and obtaining regulatory approval through prospective, task-specific clinical validation [106,136,137,141,142].

## 7. Conclusions

To move the field forward, future efforts should prioritize investigating the core dependencies of radiomics workflows—to standardize key components across the entire pipeline, from image processing to predictive modeling. Future breakthroughs in radiomics will be driven by a move toward more robust and generalizable models. This will be achieved through the integration of multi-omics data, the leveraging of foundation models, and the establishment of standardized datasets. Furthermore, techniques like federated learning and the use of synthetic data will be crucial for overcoming data-access barriers and enhancing model performance across diverse populations. Statistical validation is a necessary but not a sufficient condition for successful clinical implementation. This would require a change from the classical metrics used in academic publications (e.g., statistical significance and accuracy) towards practical measures such as feasibility, acceptability, safety and clinical utility.

Summing up, we argue that technical progress alone is insufficient, and minimum reporting standards for clinical translatability are required. It must be noted, then, that any radiomics study claiming clinical significance must be able to verify at least five aspects. First, externally validating its results on at least two datasets from different vendor types or acquisition processes, with performance measures reported separately per group. Second, providing a perturbation test that assesses how many of the extracted features and predictions persist when the acquisition process is varied within clinically acceptable ranges. Third, reporting calibration metrics (calibration curves, Brier score, and Expected Calibration Error) along with discrimination measures, with 95% confidence intervals estimated via bootstrap, and subgroup-specific calibration reported for internal and external cohorts. Fourth, performing faithfulness testing on any form of interpretability method used in the study. Fifth, reporting all the relevant acquisition parameters and possible confounders due to demographic differences in the training versus validation dataset.

To further strengthen the reliability and generalizability of radiomics-based models, we encourage researchers to adopt rigorous validation protocols that include patient-level data splitting, pre-registration of analysis plans, external validation on independent multi-institutional cohorts, and transparent reporting of confidence intervals and calibration metrics. Journals and reviewers should enforce these standards to distinguish between engineering prototypes and truly clinically ready systems.

We are not suggesting that those studies that do not fulfill these criteria should be discarded—such exploration is necessary. But what we are suggesting is that no study should use phrases like “clinically ready,” “deployable,” and “real-world validated” unless it satisfies all five criteria. This can easily be adopted as a condition by journals for their reviewers to draw a line between an engineering prototype and a true translational system. In the absence of minimum criteria, the field is destined to develop algorithms that work in ideal conditions but fail in the clinical setting.

## Figures and Tables

**Table 1:** Exemplary studies using traditional machine learning and hand-crafted radiomic features in oncologic imaging. For each study, we report sample size and external validation status as key indicators of generalizability. Performance metrics are reported as originally published and are not directly comparable across studies (see Section 2).

Reference	Imaging Modality	Sample Size	External Validation	Used Method	Obtained Result	Remaining Challenge
Parmar et al. (2015) [11]	CT	464 patients (training: 310, validation: 154)	Yes (two independent cohorts)	RF, SVM, multiple classifiers	RF with Wilcoxon feature selection achieved the highest prognostic performance for NSCLC survival prediction (AUC ≈ 66%).	Pipeline sensitivity and reproducibility.
Zheng et al. (2022) [13]	CT (multiphase)	388 patients (training: 272, test: 116)	Yes (two independent cohorts)	LR, SVM, RF	SVM achieved the best radiomics-model performance (AUC: 0.844 training; 0.840 test). Combined model (radiomics + clinical) achieved the best overall performance (AUC: 0.904 training; 0.854 test).	Limited single-center validation.
Li et al. (2024) [14]	DCE-MRI (T1-weighted)	281 patients (training: 70%, validation: 30%)	No (single dataset, random split 7:3)	SVM, RF, DT, KNN, XGBoost, LightGBM	LightGBM achieved the best overall performance for pCR prediction (AUC: 0.823). RF performed best in the luminal subtype (AUC: 0.914); LightGBM performed best in the triple-negative subtype (AUC: 0.836).	Generalizability across external datasets not evaluated.
Tang et al. (2023) [15]	CT	422 patients (TCIA dataset, NSCLC)	No (70:30 cross-validation)	DT, BT, RF, SVM, GLM, DL-ANN	RF achieved the best overall performance (AUC: 0.938). BT achieved AUC: 0.912. DL-ANN showed no significant advantage over traditional ML (AUC: 0.705).	Limited robustness due to single-dataset validation (TCIA).
Ma et al. (2024) [17]	CT + MRI (multiphase)	221 patients (97 HCC, 124 non-HCC)	No (single-center, 5-fold CV)	SVM, KNN, RF, XGBoost, LightGBM, MLP	Combined CT+MR radiomics-clinical model achieved the best diagnostic performance for hepatocellular carcinoma (HCC) classification (accuracy: 82.4%, SVM).	External validation not performed.
Shayesteh et al. (2021) [18]	T2-weighted MRI	53 patients (training Center#1: 36; validation Center#2: 17)	Yes (two centers)	KNN, NB, RF, XGBoost	Delta-radiomic RF model achieved the highest performance (AUC: 0.96 ± 0.01), followed by NB (AUC: 0.96 ± 0.04).	Limited external validation cohort size (*n* = 17).
Xie et al. (2025) [19]	Contrast-enhanced T1-weighted MRI	180 patients (118 lung, 62 breast; training: 126; validation: 54)	No (single-center, random split 7:3)	LR, SVM, KNN, MLP, LightGBM	LightGBM achieved the best performance (AUC: 0.875 training; 0.866 validation).	External validation on independent datasets not performed; retrospective single-center design.

**Table 2:** Exemplary studies using CNN-based deep learning radiomics in oncologic imaging. For each study, we report sample size and external validation status as key indicators of generalizability. Performance metrics are reported as originally published and are not directly comparable across studies (see Section 2).

Reference	Imaging Modality	Sample Size	External Validation	Used Method	Obtained Result	Remaining Challenge
Mahmud et al. (2023) [30]	MRI	3264 MR images	No (single dataset, split into 80% training, 10% validation, and 10% test).	Custom CNN, ResNet50, VGG16, InceptionV3	Custom CNN achieved the best brain tumor detection performance (accuracy: 93.3%; AUC: 98.4%).	Limited generalizability assessment.
Yang et al. (2025) [31]	MRI	293 patients (128 LGT, 165 HGT)	No (single dataset, split into 80% training and 20% validation)	BrainCNN, MobileNet, InceptionV3, ResNet50	BrainCNN achieved the best low- vs. high-grade brain tumor classification performance (accuracy: 99.5%).	Potential overfitting due to limited dataset size.
Zhang et al. (2024) [32]	CT	660 patients from 2 large medical centers	Yes (528 patients for training and 132 patients for external test)	3D-CNN	3D-CNN achieved the best EGFR mutation prediction performance, outperforming radiomic and clinical models (AUC: 94.7%).	Acquisition variability may affect reproducibility.
Chaudhary et al. (2026) [33]	MRI	2146 MRI images	No (single dataset, split into 70% training, 20% validation, and 10% test)	U-Net, SSD	U-Net achieved the best brain tumor segmentation performance (accuracy: 97.7%), substantially outperforming SSD (58%).	Limited external validation.
Yu et al. (2025) [34]	CT	1429 patients; 1626 nodules	No (single-center dataset, split into 80% training and 20% validation	RNN, U-Net, WFCM, SNAKE	RNN-based segmentation achieved the best performance (Dice: 80.3%) with strong downstream diagnostic results.	Sensitivity to contour and imaging variability.
Devadas et al. (2026) [35]	MRI	The Figshare Brain Tumor MRI Dataset (3064 MRI scans (80% training, 10% validation, and 10% test))	Yes (the Harvard Whole Brain Atlas dataset)	Mask R-CNN + GLCM radiomics + MLP	Hybrid framework achieved the best brain tumor detection performance (classification accuracy: 0.982; localization accuracy: 99%).	Reduced interpretability and computational efficiency.
Chen et al. (2023) [36]	DCE-MRI + DWI	488 lesions of 479 patients	No (single dataset, 366 lesions for training cohort and 122 lesions for testing cohort)	Deep learning radiomics nomogram	Combined deep radiomics nomogram achieved the best axillary lymph node metastasis prediction performance (training AUC: 80%; testing AUC: 71%).	Generalization challenges across datasets.
Khanfari et al. (2023) [37]	Multiparam MRI	The PROSTATEx-2 dataset (111 patients)	No (Single dataset, 5-fold CV)	CNN deep features + PCA + SVM	Combined deep-radiomic model achieved the best prostate cancer Gleason grading performance (AUC: 94%).	Increased complexity and overfitting risk.
Qureshi et al. (2023) [38]	Multiparam MRI	The BRATS-2021 dataset (585 patients)	No (single dataset, 10-fold CV)	CNN latent features + GLCM + HOG + LBP	Radiogenomic framework achieved the best MGMT methylation prediction performance (accuracy: 96.8%).	Computational complexity and limited interpretability.
Cho et al. (2024) [39]	Sequential MRI	194 patients (369 lesions; 776 MRI exams)	Yes (43 patients (62 lesions; 172 MRI exams))	Radiomics, CNN, Conv-GRU	Conv-GRU achieved the best brain metastasis treatment response prediction performance (developmental AUC: 87.8%; external AUC: 83.4%).	Dependence on longitudinal imaging data.

**Table 3:** Exemplary studies using attention mechanisms and multimodal learning in oncologic imaging. For each study, we report sample size and external validation status as key indicators of generalizability. Performance metrics are reported as originally published and are not directly comparable across studies (see Section 2).

Reference	Imaging Modality	Sample Size	External Validation	Used Method	Obtained Result	Remaining Challenge
Zhou et al. (2023) [50]	Multipar. MRI	267 patients from SAHSYU (193 for training, 74 for internal validation)	Yes (155 patients from ZCH)	Channel- attention fusion	AUC: 89.8% (internal) and 87.3% (external) for nCRT response prediction.	Limited modality availability in clinical practice.
Nishizawa et al. (2025) [51]	Longitudinal MRI + clinical	660 patients from the I-SPY 2 dataset (5-fold CV (528 for training, 132 for validation))	Yes (114 patients from the I-SPY 1 dataset)	3D CNN + self-attention	AUC: 73% (internal) and 71% (external) for pCR prediction in breast cancer.	Limited cross-institutional generalizability.
Zhang et al. (2025) [52]	MRI + pathology + clinical	363 patients from SCWCH hospital (290 for training and 73 for validation)	Yes (204 patients from CMUSJH hospital)	Vision Transformer + co-attention	MuFi achieved AUCs of 90.2%, 81.8%, and 81.6%. in training, validation, and external cohorts, respectively.	Limited multicenter validation.
Sarwar et al. (2025) [53]	MRI + genomics	206 paired MRI-genomic samples (TCGA-GBM) and 293 (TCGA-LGG)	No (85%/15% train–test split)	Graph Attention Networks + cross-modal attention	MDL-CA achieved accuracies ranging from 96.22% to 98.46% across benchmark datasets.	Dependence on complete multimodal data.
Gan et al. (2025) [54]	CT + pathomics	118 patients from center 1 (83 for training and 35 for validation)	Yes (153 patients from centers 2 and 3)	Transformer attention fusion	External AUC of 85.8% for major pathological response prediction in NSCLC.	Uncertain generalizability across larger cohorts.
Ji et al. (2026) [55]	Ultrasound	3156 patients across 8 centers (70%/30% internal train–test split)	Yes (756 patients as external test cohort)	Vision Transformer + TabTransformer + radiomics	Training AUC of 98% and external AUC of 95.8% across eight institutions (3156 patients).	High model complexity and uncertain transferability.

**Table 4:** Exemplary studies using foundation models and self-supervised learning in oncologic imaging. For each study, we report sample size and external validation status as key indicators of generalizability. Performance metrics are reported as originally published and are not directly comparable across studies (see Section 2).

Reference	Imaging Modality	Sample Size	External Validation	Used Method	Obtained Result	Remaining Challenge
Pai et al. (2024) [66]	CT	291 patients from the HarvardRT dataset	Yes (420 patients from LUNG1 and 133 patients from RADIO)	Oncology foundation model	AUC of 94.4% for lung nodule malignancy prediction; AUCs of 63.8% and 65.3% for 2-year NSCLC survival prediction on two external cohorts.	Pretraining data from academic centers; potential distribution bias at deployment.
Chen et al. (2024) [67]	Histopathology	100,426 WSIs (pretraining); 34 downstream tasks	Yes (CPTAC, EBRAINS, DHMC, CHA, HEL)	UNI (100M-image pretraining)	AUC of 97.6% for 43-class cancer-type classification; average improvement of 8.3–10.0% across 15 downstream slide-level tasks over strong baselines.	Limited to histopathology; cross-modality generalization not evaluated.
Huang et al. (2026) [68]	Longitudinal MRI	3928 MRIs from 1339 patients (529 for training, 133 for internal validation, and 677 for external validation)	Yes (381 patients from center 2 and 296 patients from center 3)	BSTNet (self-supervised temporal learning)	AUCs of 88.2%, 85.7%, and 85.4% across internal and two external validation cohorts for breast cancer risk prediction.	Single cancer application; broader transferability unclear.
Missaoui et al. (2026) [69]	Brain MRI	4456 MRI scans (15-class dataset) and 7023 MRI scans (4-class dataset)	No (internal 5-fold CV only)	DINO v1/v2/v3 + lightweight classifiers	Accuracy of 98.17% on a 15-class and 99.08% on a 4-class brain tumor classification dataset.	Further validation on heterogeneous clinical datasets warranted.
Renugadevi et al. (2025) [70]	Multimodal MRI	237 glioma patients (BraTS 2020)	Yes (210 glioma patients (BraTS 2019))	SimCLR + nnU-Net + radiomics + clinical data	C-index of 87% (internal) and 86% (external) for glioblastoma survival prediction.	Evaluated primarily on BraTS datasets; limited clinical population diversity.
Li et al. (2026) [71]	CT	33,901 CT scans; 656 patients	Yes (TCGA-LUSC)	UCLIF (contrastive masked image modeling + ViT)	AUC up to 97% for survival prediction and 96% for lung cancer classification across multicenter datasets.	Pretraining limited to chest CT; cross-cancer transferability unclear.
Yang et al. (2025) [72]	CT	5137 CT images (pretraining); 1195 CT images (fine-tuning/validation, 5-fold CV)	Yes (120 CRC patients, Ningbo Medical Center Lihuili Hospital)	CRCFound (MAE-based foundation model)	AUCs of 88.9–95.2% for TNM staging, MSI prediction, and survival analysis in colorectal cancer.	Developed and validated only for colorectal cancer; broader transferability unclear.
Li et al. (2026) [73]	Multimodal Brain MRI	51,029 brain MRIs (40,823 for training and 10,206 for testing)	Yes (Burdenko-GBM-Progression, TCGA-LGG)	UMBIF (masked autoencoding foundation model)	AUCs of 91.6%, 89.6%, 85.9%, and 81.5% for molecular marker prediction and progression assessment across multicenter datasets.	Restricted to brain MRI; broader anatomical transferability not evaluated.
Gomaa et al. (2025) [74]	MRI + clinical + RT planning	2317 MRI studies (BraTS 2021, UPenn-GBM, UCSF-PDGM; pretraining); 59 patients from Burdenko dataset (training, 5-fold CV)	Yes (20 patients from GlioCMV UKER dataset, University Hospital Erlangen)	Self-supervised multimodal transformer	External AUC of 75.3% for pseudoprogression vs. true progression distinction in glioblastoma.	External performance remains moderate; labeled cohort size limited.

## Data Availability

No new data were created or analyzed in this study.

## References

[1] Samala R.K., Drukker K., Shukla-Dave A., Chan H.P., Sahiner B., Petrick N., Greenspan H., Mahmood U., Summers R.M., Tourassi G. (2024). AI and machine learning in medical imaging: Key points from development to translation. Br. J. Radiol. Artif. Intell..

[2] Jiang X., Hu Z., Wang S., Zhang Y. (2023). Deep learning for medical image-based cancer diagnosis. Cancers.

[3] Emran A.S.H., Akter H., Shiam A.A. (2025). Brain Tumor MRI Dataset. https://data.mendeley.com/datasets/c9rt8d6zrf/1.

[4] Xu Y., Zheng B., Liu X., Wu T., Ju J., Wang S., Lian Y., Zhang H., Liang T., Sang Y. (2022). Annotated Ultrasound Liver Images. Dataset. https://zenodo.org/records/7272660.

[5] JTIPTJ (2022). Chest X-Ray (Pneumonia, COVID-19, Tuberculosis). https://www.kaggle.com/datasets/jtiptj/chest-xray-pneumoniacovid19tuberculosis.

[6] McNatt G. (2023). Multi-Modality Brain Tumor MRI & PET DICOM Series. https://www.kaggle.com/datasets/grantmcnatt/mri-and-pet-dice-similarity-dataset.

[7] Zhao B., Schwartz L.H., Kris M.G., Riely G.J. (2015). Coffee-Break Lung CT Collection with Scan Images Reconstructed at Multiple Imaging Parameters. https://www.cancerimagingarchive.net/collection/rider-lung-ct/.

[8] Rogers W., Thulasi Seetha S., Refaee T.A., Lieverse R.I., Granzier R.W., Ibrahim A., Keek S.A., Sanduleanu S., Primakov S.P., Beuque M.P. (2020). Radiomics: From qualitative to quantitative imaging. Br. J. Radiol..

[9] Zhao B. (2021). Understanding and managing sources of variation in radiomics. Front. Oncol..

[10] Teng X., Wang Y., Nicol A.J., Ching J.C.F., Wong E.K.Y., Lam K.T.C., Zhang J., Lee S.W.Y., Cai J. (2024). Enhancing the clinical utility of radiomics: Addressing the challenges of repeatability and reproducibility in CT and MRI. Diagnostics.

[11] Parmar C., Grossmann P., Bussink J., Lambin P., Aerts H.J. (2015). Machine learning methods for quantitative radiomic biomarkers. Sci. Rep..

[12] Zhang H.-w., Wang Y.-r., Hu B., Song B., Wen Z.-j., Su L., Chen X.-m., Wang X., Zhou P., Zhong X.-m. (2024). Using machine learning to develop a stacking ensemble learning model for the CT radiomics classification of brain metastases. Sci. Rep..

[13] Zheng Y., Zhou D., Liu H., Wen M. (2022). CT-based radiomics analysis of different machine learning models for differentiating benign and malignant parotid tumors. Eur. Radiol..

[14] Li X., Li C., Wang H., Jiang L., Chen M. (2024). Comparison of radiomics-based machine-learning classifiers for the pretreatment prediction of pathologic complete response to neoadjuvant therapy in breast cancer. PeerJ.

[15] Tang F.h., Xue C., Law M.Y., Wong C.y., Cho T.h., Lai C.k. (2023). Prognostic prediction of cancer based on radiomics features of diagnostic imaging: The performance of machine learning strategies. J. Digit. Imaging.

[16] Clark K., Vendt B., Smith K., Freymann J., Kirby J., Koppel P., Moore S., Phillips S., Maffitt D., Pringle M. (2013). The Cancer Imaging Archive (TCIA): Maintaining and operating a public information repository. J. Digit. Imaging.

[17] Ma Y., Gong Y., Qiu Q., Ma C., Yu S. (2024). Research on multi-model imaging machine learning for distinguishing early hepatocellular carcinoma. BMC Cancer.

[18] Shayesteh S., Nazari M., Salahshour A., Sandoughdaran S., Hajianfar G., Khateri M., Yaghobi Joybari A., Jozian F., Fatehi Feyzabad S.H., Arabi H. (2021). Treatment response prediction using MRI-based pre-, post-, and delta-radiomic features and machine learning algorithms in colorectal cancer. Med. Phys..

[19] Xie Y., Li X., Yang S., Jia F., Han Y., Huang M., Chen L., Zou W., Deng C., Liang Z. (2025). Radiomics models using machine learning algorithms to differentiate the primary focus of brain metastasis. Transl. Cancer Res..

[20] Tabassum M., Suman A.A., Suero Molina E., Pan E., Di Ieva A., Liu S. (2023). Radiomics and machine learning in brain tumors and their habitat: A systematic review. Cancers.

[21] Piedimonte S., Mohamed M., Rosa G., Gerstl B., Vicus D. (2025). Predicting response to treatment and survival in advanced ovarian cancer using machine learning and radiomics: A systematic review. Cancers.

[22] Zwanenburg A., Vallières M., Abdalah M.A., Aerts H.J., Andrearczyk V., Apte A., Ashrafinia S., Bakas S., Beukinga R.J., Boellaard R. (2020). The image biomarker standardization initiative: Standardized quantitative radiomics for high-throughput image-based phenotyping. Radiology.

[23] Orlhac F., Eertink J.J., Cottereau A.S., Zijlstra J.M., Thieblemont C., Boellaard R. (2022). A guide to ComBat harmonization of imaging biomarkers in multicenter studies. J. Nucl. Med..

[24] Zhang J., Teng X., Zhang X., Lam S.K., Lin Z., Liang Y., Yu H., Siu S.W.K., Chang A.T.Y., Zhang H. (2023). Comparing effectiveness of image perturbation and test retest imaging in improving radiomic model reliability. Sci. Rep..

[25] Zwanenburg A., Leger S., Agolli L., Pilz K., Troost E.G., Richter C., Löck S. (2019). Assessing robustness of radiomic features by image perturbation. Sci. Rep..

[26] Vrettos K., Triantafyllou M., Marias K., Karantanas A.H., Klontzas M.E. (2024). Artificial intelligence-driven radiomics: Developing valuable radiomics signatures with the use of artificial intelligence. Br. J. Radiol. Artif. Intell..

[27] Demircioğlu A. (2025). Reproducibility and interpretability in radiomics: A critical assessment. Diagn. Interv. Radiol..

[28] Buvat I., Dutta J., Jha A.K., Siegel E., Yousefirizi F., Rahmim A., Bradshaw T. (2025). Should end-to-end deep learning replace handcrafted radiomics?. Eur. J. Nucl. Med. Mol. Imaging.

[29] Gao S., Liu J., Li L., Yang D., Miao Y., Zhang X., Han Q., Shi Y., Wu J., Zhang K. (2026). Application of deep learning technology in breast cancer: A systematic review of segmentation, detection, and classification approaches. BioMed. Eng. OnLine.

[30] Mahmud M.I., Mamun M., Abdelgawad A. (2023). A deep analysis of brain tumor detection from mr images using deep learning networks. Algorithms.

[31] Yang J., Siddique M.A., Ullah H., Gilanie G., Por L.Y., Alshathri S., El-Shafai W., Aldossary H., Gadekallu T.R. (2025). Braincnn: Automated brain tumor grading from magnetic resonance images using a convolutional neural network-based customized model. SLAS Technol..

[32] Zhang G., Shang L., Cao Y., Zhang J., Li S., Qian R., Liu H., Zhang Z., Pu H., Man Q. (2024). Prediction of epidermal growth factor receptor (EGFR) mutation status in lung adenocarcinoma patients on computed tomography (CT) images using 3-dimensional (3D) convolutional neural network. Quant. Imaging Med. Surg..

[33] Chaudhary R., Chaudhary P., Singh C., Kumar K., Singh S., Arora R., Kaur S., Vaarshney D., Acharya P., Mishra U. (2026). Automated Brain Tumor Detection Using Convolutional Neural Network. Biotechnol. Appl. Biochem..

[34] Yu Y., Li G.F., Tan W.X., Qu X.Y., Zhang T., Hou X.Y., Zhu Y.B., Ma Z.Y., Yang L., Gao Y. (2025). Towards automatical tumor segmentation in radiomics: A comparative analysis of various methods and radiologists for both region extraction and downstream diagnosis. BMC Med. Imaging.

[35] Devadas P., Mathivanan G. (2026). Mask-Region-based Convolutional Neural Networks (R-CNN) with Radiomics Integration and Gray Level Co-occurrence Matrix (GLCM) for brain tumor detection and segmentation. PLoS ONE.

[36] Chen Y., Wang L., Dong X., Luo R., Ge Y., Liu H., Zhang Y., Wang D. (2023). Deep learning radiomics of preoperative breast MRI for prediction of axillary lymph node metastasis in breast cancer. J. Digit. Imaging.

[37] Khanfari H., Mehranfar S., Cheki M., Mohammadi Sadr M., Moniri S., Heydarheydari S., Rezaeijo S.M. (2023). Exploring the efficacy of multi-flavored feature extraction with radiomics and deep features for prostate cancer grading on mpMRI. BMC Med. Imaging.

[38] Qureshi S.A., Hussain L., Ibrar U., Alabdulkreem E., Nour M.K., Alqahtani M.S., Nafie F.M., Mohamed A., Mohammed G.P., Duong T.Q. (2023). Author Correction: Radiogenomic classification for MGMT promoter methylation status using multi-omics fused feature space for least invasive diagnosis through mpMRI scans. Sci. Rep..

[39] Cho S.J., Cho W., Choi D., Sim G., Jeong S.Y., Baik S.H., Bae Y.J., Choi B.S., Kim J.H., Yoo S. (2024). Prediction of treatment response after stereotactic radiosurgery of brain metastasis using deep learning and radiomics on longitudinal MRI data. Sci. Rep..

[40] Salmanpour M.R., Mehrnia S.S., Jabarzadeh Ghandilu S., Safahi Z., Falahati S., Taeb S., Mousavi G., Maghsudi M., Shariftabrizi A., Hacihaliloglu I. (2026). Handcrafted vs. Deep Radiomics vs. Fusion vs. Deep Learning: A Comprehensive Review of Machine Learning-Based Cancer Outcome Prediction in PET and SPECT Imaging. J. Imaging Inform. Med..

[41] Yao I.Z., Dong M., Hwang W.Y. (2025). Deep learning applications in clinical cancer detection: A review of implementation challenges and solutions. Mayo Clin. Proc. Digit. Health.

[42] Piffer S., Ubaldi L., Tangaro S., Retico A., Talamonti C. (2024). Tackling the small data problem in medical image classification with artificial intelligence: A systematic review. Prog. Biomed. Eng..

[43] Rundo L., Militello C. (2024). Image biomarkers and explainable AI: Handcrafted features versus deep learned features. Eur. Radiol. Exp..

[44] Garg P., Sharma M., Kumar P. (2025). Transparency in diagnosis: Unveiling the power of deep learning and explainable AI for medical image interpretation. Arab. J. Sci. Eng..

[45] Lilhore U.K., Sunder R., Simaiya S., Alsafyani M., Monish Khan M., Alroobaea R., Alsufyani H., Baqasah A.M. (2025). AG-MS3D-CNN multiscale attention guided 3D convolutional neural network for robust brain tumor segmentation across MRI protocols. Sci. Rep..

[46] Li Y., Shi J., Wang Y., Wei J., Wei Y., Wu L., Wang M., Pan Z. (2026). DBMAF: Dual-branch multimodal attention-based feature fusion network for fusing histopathology and radiology images. Biomed. Signal Process. Control.

[47] Dao D.P., Yang H.J., Kim S.H., Kang S.R. (2026). AttCo: Attention-based Co-Learning Fusion of Deep Feature Representation for Medical Image Segmentation using Multimodality. Neural Netw..

[48] Hossain K.F., Kamran S.A., Ong J., Tavakkoli A. (2025). Enhancing efficient deep learning models with multimodal, multi-teacher insights for medical image segmentation. Sci. Rep..

[49] Al-Zoghby A.M., Ebada A.I., Saleh A.S., Abdelhay M., Awad W.A. (2025). A comprehensive review of multimodal deep learning for enhanced medical diagnostics. Comput. Mater. Contin..

[50] Zhou X., Yu Y., Feng Y., Ding G., Liu P., Liu L., Ren W., Zhu Y., Cao W. (2023). Attention mechanism based multi-sequence MRI fusion improves prediction of response to neoadjuvant chemoradiotherapy in locally advanced rectal cancer. Radiat. Oncol..

[51] Nishizawa T., Maldjian T., Jiao Z., Duong T.Q. (2025). Attention-based multimodal deep learning for interpretable and generalizable prediction of pathological complete response in breast cancer. J. Transl. Med..

[52] Zhang W., Zhang S., You J., Li F., Wu X., Lu X., Lv Q., Huang J., Yi Y., Bu H. (2025). Attention-based multimodal fusion transformer for predicting the efficacy of neoadjuvant therapy in breast cancer: A cross-institutional retrospective study. Breast Cancer Res..

[53] Sarwar S., Majeed S., Nawaz A., Bibi R., Lee S.W. (2025). MDL-CA: A multimodal deep learning approach with a cross attention mechanism for accurate brain cancer diagnosis. Front. Public Health.

[54] Gan X., He J., Zhang W., Chen W., Liu S., Li W., Duan X., Lv L., Liang Y., Cao Q. (2025). Attention-guided framework for integrative omics and temporal dynamics in predicting major pathological response in neoadjuvant immunochemotherapy for NSCLC. J. Immunother. Cancer.

[55] Ji X., Liu C., Hu J., Li S., Wang L., Cheng X., Liu C., Zhang Y. (2026). A multicenter deep learning framework integrating radiomics and vision transformers for comprehensive ovarian tumor analysis from ultrasound imaging. Eur. J. Med. Res..

[56] Zhang B., Wan Z., Luo Y., Zhao X., Samayoa J., Zhao W., Wu S. (2025). Multimodal integration strategies for clinical application in oncology. Front. Pharmacol..

[57] Yang H., Yang M., Chen J., Yao G., Zou Q., Jia L. (2025). Multimodal deep learning approaches for precision oncology: A comprehensive review. Brief. Bioinform..

[58] Rai N., Dahal P.R. (2026). A Unified Attention U-Net Framework for Cross-Modality Tumor Segmentation in MRI and CT. arXiv.

[59] Chung M., Won J.B., Kim G., Kim Y., Ozbulak U. (2024). Evaluating visual explanations of attention maps for transformer-based medical imaging. Medical Image Computing and Computer Assisted Intervention—MICCAI 2024 Workshops.

[60] Xu J., Zhuang S., He Y., Wang H., Zhuang Z., Zeng H. (2026). Multimodal Sparse Fusion Transformer Network with Spatio-Temporal Decoupling for Breast Tumor Classification. Med. Image Anal..

[61] Hong T., Huang W., Lu W., Peng L., Miao C., Chen L., Yang Y., Lin Y., Wu L. (2026). A dual-center study: Multimodal fusion-based deep learning approach for pathological subtype prediction of type I and type II ovarian cancer. BMC Med. Imaging.

[62] Paschali M., Chen Z., Blankemeier L., Varma M., Youssef A., Bluethgen C., Langlotz C., Gatidis S., Chaudhari A. (2025). Foundation models in radiology: What, how, why, and why not. Radiology.

[63] van Veldhuizen V., Botha V., Lu C., Cesur M.E., Lipman K.G., de Jong E.D., Horlings H., Sanchez C.I., Snoek C.G., Wessels L. (2025). Foundation Models in Medical Imaging: A Review and Outlook. arXiv.

[64] Fang M., Wang Z., Pan S., Feng X., Zhao Y., Hou D., Wu L., Xie X., Zhang X.Y., Tian J. (2025). Large models in medical imaging: Advances and prospects. Chin. Med. J..

[65] Qu L., Zhang C., Hou Y., Tang F., Sheng W., Huang D., Song Z. (2026). Foundation Model-Enabled Multimodal Deep Learning for Prognostic Prediction in Colorectal Cancer with Incomplete Modalities: A Multi-Institutional Retrospective Study. Adv. Sci..

[66] Pai S., Bontempi D., Hadzic I., Prudente V., Sokač M., Chaunzwa T.L., Bernatz S., Hosny A., Mak R.H., Birkbak N.J. (2024). Foundation model for cancer imaging biomarkers. Nat. Mach. Intell..

[67] Chen R.J., Ding T., Lu M.Y., Williamson D.F.K., Jaume G., Song A.H., Chen B., Zhang A., Shao D., Shaban M. (2024). Towards a general-purpose foundation model for computational pathology. Nat. Med..

[68] Huang X., Xu Z., Zhao Y., Wang Y., Liu Y., Hu W., Zhao K., Yao L., He J., Yu Y. (2026). Longitudinal MRI-based deep learning model for predicting pathological complete response in breast cancer: A multicenter, retrospective cohort study. npj Precis. Oncol..

[69] Missaoui R., Del Coco M., Saadaoui W., Hechkel W., Helali A., Carcagnì P., Leo M. (2026). Brain Tumor Classification Using DINO Features and Lightweight Classifiers. Electronics.

[70] Renugadevi M., Ramkumar K., Raju N., Adalarasu K., Prasath S., Narasimhan K. (2025). Self-Supervised DRL with Twin Critics: A Novel Framework for Glioblastoma Survival Prognostication. Int. J. Imaging Syst. Technol..

[71] Li J., Xing Y., Gao X., Ye Z., Wang M., Song F. (2026). A Self-Supervised Foundation Model Based on Three-Dimensional Chest CT Scans for Lung Cancer Diagnosis and Prognosis Prediction. Radiol. Imaging Cancer.

[72] Yang J., Cai D., Liu J., Zhuang Z., Zhao Y., Wang F.a., Li C., Hu C., Gai B., Chen Y. (2025). CRCFound: A Colorectal Cancer CT Image Foundation Model Based on Self-Supervised Learning. Adv. Sci..

[73] Li J., Liu R., Xing Y., Gao X., Yin Q., Su Q. (2026). Foundation Model Based on Routine Magnetic Resonance Imaging for Brain Tumor Molecular Profiling and Progression Prediction. JCO Precis. Oncol..

[74] Gomaa A., Huang Y., Stephan P., Breininger K., Frey B., Dörfler A., Schnell O., Delev D., Coras R., Donaubauer A.J. (2025). A self-supervised multimodal deep learning approach to differentiate post-radiotherapy progression from pseudoprogression in glioblastoma. Sci. Rep..

[75] D’Antonoli T.A., Bluethgen C., Cuocolo R., Klontzas M.E., Ponsiglione A., Kocak B. (2025). Foundation models for radiology: Fundamentals, applications, opportunities, challenges, risks, and prospects. Diagn. Interv. Radiol..

[76] Phuntsho K., Abdullah, Lee K., Lee I., Ahn E. (2025). Adaptation of Foundation Models for Medical Image Analysis: Strategies, Challenges, and Future Directions. arXiv.

[77] Thiringer E., Gustafsson F.K., Eriksson K.L., Rantalainen M. (2026). Scanner-Induced Domain Shifts Undermine the Robustness of Pathology Foundation Models. arXiv.

[78] de Almeida J.G., Alberich L.C., Tsakou G., Marias K., Tsiknakis M., Lekadir K., Marti-Bonmati L., Papanikolaou N. (2025). Foundation models for radiology—the position of the AI for Health Imaging (AI4HI) network. Insights Imaging.

[79] Kaczmarek E., Szeto J., Nichyporuk B., Arbel T. Building a General SimCLR Self-Supervised Foundation Model Across Neurological Diseases to Advance 3D Brain MRI Diagnoses. Proceedings of the IEEE/CVF International Conference on Computer Vision.

[80] Azizi S., Culp L., Freyberg J., Mustafa B., Baur S., Kornblith S., Chen T., Tomasev N., Mitrović J., Strachan P. (2023). Robust and data-efficient generalization of self-supervised machine learning for diagnostic imaging. Nat. Biomed. Eng..

[81] Zhuang J., Luo L., Wang Q., Wu M., Luo L., Chen H. (2025). Advancing volumetric medical image segmentation via global-local masked autoencoders. IEEE Trans. Med. Imaging.

[82] Truhn D., Eckardt J.N., Ferber D., Kather J.N. (2024). Large language models and multimodal foundation models for precision oncology. npj Precis. Oncol..

[83] Mishra V., Lotter W. (2025). Comparing Computational Pathology Foundation Models using Representational Similarity Analysis. arXiv.

[84] Singh Y., Hathaway Q.A., Keishing V., Salehi S., Wei Y., Horvat N., Vera-Garcia D.V., Choudhary A., Mula Kh A., Quaia E. (2025). Beyond post hoc explanations: A comprehensive framework for accountable AI in medical imaging through transparency, interpretability, and explainability. Bioengineering.

[85] Mali S.A., Rad N.M., Woodruff H.C., Depeursinge A., Andrearczyk V., Lambin P. (2025). Harmonizing CT scanner acquisition variability in an anthropomorphic phantom: A comparative study of image-level and feature-level harmonization using GAN, ComBat, and their combination. PLoS ONE.

[86] Mühlberg A., Katzmann A., Heinemann V., Kärgel R., Wels M., Taubmann O., Lades F., Huber T., Maurus S., Holch J. (2020). The technome-a predictive internal calibration approach for quantitative imaging biomarker research. Sci. Rep..

[87] Liu Y., Wang G., Yan X., Wang H., Li T., Yang S., Liu Z., Zhang S., Su X. (2025). Effect of reconstruction settings on radiomics feature robustness in positron emission tomography images: A clinical study. Quant. Imaging Med. Surg..

[88] Virani-Wall J., Peoples J., Hamghalam M., Gangai N., Gonen M., James I., Kang H., Rong X., Wasim M., Chun Y. (2025). Prospective study on the reproducibility of radiomic features in the setting of variable CT contrast timing: Initial results. Medical Imaging 2025: Computer-Aided Diagnosis.

[89] Yoon J.S., Oh K., Shin Y., Mazurowski M.A., Suk H.I. (2024). Domain generalization for medical image analysis: A review. Proc. IEEE.

[90] Madni H.A., Shujat H., Zottin S., De Nardin A., Foresti G.L. (2026). Mitigating Data Scarcity in Cancer Classification with Synthetic Data. IEEE Access.

[91] Sar A., Kumar A., Roy S., Kaushish A., Choudhury T., Minakshi, Alqahtani S.S., Abraham A. (2026). Quantum Machine Learning in Medical Image Analysis: From Diagnostics to Surgery Planning. IEEE Access.

[92] Yu H., Wang J., Chen M., Rodriguez C., Tanaka Y. (2025). Assessing the generalizability of artificial intelligence in radiology: A systematic review of performance across different clinical settings. Int. J. Surg..

[93] Yu A.C., Mohajer B., Eng J. (2022). External validation of deep learning algorithms for radiologic diagnosis: A systematic review. Radiol. Artif. Intell..

[94] Agostini A., Pescatori L., Sconfienza L.M., Albano D., Messina C. (2025). Deep learning in rib fracture imaging: Study quality assessment using the Must AI Criteria-10 (MAIC-10) checklist for artificial intelligence in medical imaging. Insights Imaging.

[95] Belue M.J., Mukhtar V., Ram R., Gokden N., Jose J., Massey J.L., Biben E., Buddha S., Langford T., Shah S. (2025). External validation of an artificial intelligence algorithm using biparametric MRI and its simulated integration with conventional PI-RADS for prostate cancer detection. Acad. Radiol..

[96] Huang X., Jacobs C., Scholten E., Prokop M. (2025). External Test of a Deep Learning Algorithm for Pulmonary Nodule Malignancy Risk Stratification Using European Screening Data. Radiology.

[97] Cuocolo R., Bernardini D., Pinto dos Santos D., Klontzas M.E., Akinci D’Antonoli T., Semedo L.C., Decoster R., Huisman M., Kotter E., Martí-Bonmatí L. (2025). AI medical device post-market surveillance regulations: Consensus recommendations by the European Society of Radiology. Insights Imaging.

[98] Kelly C.J., Karthikesalingam A., Suleyman M., Corrado G., King D. (2019). Key challenges for delivering clinical impact with artificial intelligence. BMC Med..

[99] Aydemir O. (2021). A new performance evaluation metric for classifiers: Polygon area metric. J. Classif..

[100] Haupt M., Maurer M.H., Thomas R.P. (2025). Explainable Artificial Intelligence in Radiological Cardiovascular Imaging—A Systematic Review. Diagnostics.

[101] Ahmed F., Naz N.S., Khan S., Rehman A.U., Ismael W.M., Khan M.A. (2026). Explainable artificial intelligence (XAI) in medical imaging: A systematic review of techniques, applications, and challenges. BMC Med. Imaging.

[102] Pinsky M.R., Bedoya A., Bihorac A., Celi L., Churpek M., Economou-Zavlanos N.J., Elbers P., Saria S., Liu V., Lyons P.G. (2024). Use of artificial intelligence in critical care: Opportunities and obstacles. Crit. Care.

[103] Solaiman B., Malik A. (2025). Regulating algorithmic care in the European Union: Evolving doctor–patient models through the Artificial Intelligence Act (AI-Act) and the liability directives. Med. Law Rev..

[104] Niraula D., Cuneo K.C., Dinov I.D., Gonzalez B.D., Jamaluddin J.B., Jin J.J., Luo Y., Matuszak M.M., Ten Haken R.K., Bryant A.K. (2025). Intricacies of human–AI interaction in dynamic decision-making for precision oncology. Nat. Commun..

[105] Kundu S., Pal K., Pyne A., Wang X. (2025). Force-bearing phagocytic adhesion rings mediate the phagocytosis of surface-bound particles. Nat. Commun..

[106] Friebe M. (2026). AI in radiology and interventions: A structured narrative review of workflow automation, accuracy, and efficiency gains of today and what’s coming. Int. J. Comput. Assist. Radiol. Surg..

[107] Savardi M., Signoroni A., Benini S., Vaccher F., Alberti M., Ciolli P., Di Meo N., Falcone T., Ramanzin M., Romano B. (2025). Upskilling or deskilling? Measurable role of an AI-supported training for radiology residents: A lesson from the pandemic. Insights Imaging.

[108] Nair M., Svedberg P., Larsson I., Nygren J.M. (2024). A comprehensive overview of barriers and strategies for AI implementation in healthcare: Mixed-method design. PLoS ONE.

[109] Kocak B., dos Santos D.P., Dietzel M. (2025). The widening gap between radiomics research and clinical translation: Rethinking current practices and shared responsibilities. Eur. J. Radiol. Artif. Intell..

[110] Lambin P., Woodruff H.C., Mali S.A., Zhong X., Kuang S., Lavrova E., Khan H., Lekadir K., Zwanenburg A., Deasy J. (2025). Radiomics Quality Score 2.0: Towards radiomics readiness levels and clinical translation for personalized medicine. Nat. Rev. Clin. Oncol..

[111] Strijbis V.I.J., Gurney-Champion O.J., Grama D.I., Slotman B.J., Verbakel W.F.A.R. (2025). Interpreting convolutional neural network explainability for head-and-neck cancer radiotherapy organ-at-risk segmentation. Comput. Biol. Med..

[112] Hémon C., Texier B., Lafond C., Nunes J.C., Barateau A. (2025). Towards trustworthy AI in radiotherapy: A comprehensive review of uncertainty-aware techniques. Phys. Med. Biol..

[113] Gao J., Chen M., Xiang L., Xu C. (2025). A comprehensive survey on evidential deep learning and its applications. IEEE Trans. Pattern Anal. Mach. Intell..

[114] Cheung M.Y., Veeraraghavan A., Balakrishnan G. (2026). COMPASS: Robust Feature Conformal Prediction for Medical Segmentation Metrics. arXiv.

[115] Maes A., Chen V., Thompson E., Berger D. (2025). Beyond Segmentation: Confidence-Aware and Debiased Estimation of Ratio-based Biomarkers. arXiv.

[116] Woo M., Zhang L., Brown-Mulry B., Hwang I., Gichoya J.W., Gastounioti A., Banerjee I., Seyyed-Kalantari L., Trivedi H. (2025). Subgroup evaluation to understand performance gaps in deep learning-based classification of regions of interest on mammography. PLoS Digit. Health.

[117] Vasey B., Nagendran M., Campbell B., Clifton D.A., Collins G.S., Denaxas S., Denniston A.K., Faes L., Geerts B., Ibrahim M. (2022). Reporting guideline for the early stage clinical evaluation of decision support systems driven by artificial intelligence: DECIDE-AI. Nat. Med..

[118] Reina G.A., Trask A., Howard J., White O. (2024). Federated Learning for Multi-Center Oncologic Imaging: A Prospective Study on Generalizability and Privacy Preservation. Lancet Digit. Health.

[119] National Institute for Health and Care Excellence (2019). Evidence Standards Framework for Digital Health Technologies.

[120] Bian Y., Li J., Ye C., Jia X., Yang Q. (2025). Artificial intelligence in medical imaging: From task-specific models to large-scale foundation models. Chin. Med. J..

[121] Wang F., Chen J., Zheng L., Huang X. (2026). From imaging to omics: Deep learning is bridging MRI and liquid biopsy in bone tumor diagnosis. J. Bone Oncol..

[122] Campanella G., Chen S., Singh M., Verma R., Muehlstedt S., Zeng J., Stock A., Croken M., Veremis B., Elmas A. (2025). A clinical benchmark of public self-supervised pathology foundation models. Nat. Commun..

[123] Liu B., Polack M., Coudray N., Claudio Quiros A., Sakellaropoulos T., Le H., Karimkhan A., Crobach A.S., van Krieken J.H.J., Yuan K. (2025). Self-supervised learning reveals clinically relevant histomorphological patterns for therapeutic strategies in colon cancer. Nat. Commun..

[124] Almubarak H.A. (2025). Self-supervised learning for data augmentation in histopathology image segmentation. Sci. Rep..

[125] Tayebi Arasteh S., Misera L., Kather J.N., Truhn D., Nebelung S. (2024). Enhancing diagnostic deep learning via self-supervised pretraining on large-scale, unlabeled non-medical images. Eur. Radiol. Exp..

[126] Dorfner F.J., Patel J.B., Kalpathy-Cramer J., Gerstner E.R., Bridge C.P. (2025). A review of deep learning for brain tumor analysis in MRI. npj Precis. Oncol..

[127] Oda M. (2025). Generative AI and foundation models in medical image: M. Oda. Radiol. Phys. Technol..

[128] Azad M., Fahad N.M., Raiaan M.A.K., Anik T.R., Khan M.F.K., Toyé H.M.K., Muhammad G. (2026). A Systematic Review of Diffusion Models for Medical Image-Based Diagnosis: Methods, Taxonomies, Clinical Integration, Explainability, and Future Directions. Diagnostics.

[129] Bonney L., Kalisvaart G., van Velden F., Bradley K., Hassan A., Grootjans W., McGowan D. (2025). Deep learning image enhancement algorithms in PET/CT imaging: A phantom and sarcoma patient radiomic evaluation. Eur. J. Nucl. Med. Mol. Imaging.

[130] Ruff C., Hauser T.K., Roder C., Feucht D., Bombach P., Zerweck L., Staber D., Paulsen F., Ernemann U., Gohla G. (2025). Multidisciplinary, Clinical Assessment of Accelerated Deep-Learning MRI Protocols at 1.5 T and 3 T After Intracranial Tumor Surgery and Their Influence on Residual Tumor Perception. Diagnostics.

[131] Fallahpoor M., Chakraborty S., Pradhan B., Faust O., Barua P.D., Chegeni H., Acharya R. (2024). Deep learning techniques in PET/CT imaging: A comprehensive review from sinogram to image space. Comput. Methods Programs Biomed..

[132] Zheng X., Huang Y., Lin Y., Zhu T., Zou J., Wang S., Wang K. (2023). 18F-FDG PET/CT-based deep learning radiomics predicts 5-years disease-free survival after failure to achieve pathologic complete response to neoadjuvant chemotherapy in breast cancer. EJNMMI Res..

[133] Yu X., He L., Wang Y., Dong Y., Song Y., Yuan Z., Yan Z., Wang W. (2023). A deep learning approach for automatic tumor delineation in stereotactic radiotherapy for non-small cell lung cancer using diagnostic PET-CT and planning CT. Front. Oncol..

[134] Zhong Y., Cai C., Chen T., Gui H., Deng J., Yang M., Yu B., Song Y., Wang T., Sun X. (2023). PET/CT based cross-modal deep learning signature to predict occult nodal metastasis in lung cancer. Nat. Commun..

[135] Huang J., Xiang Y., Gan S., Wu L., Yan J., Ye D., Zhang J. (2025). Application of artificial intelligence in medical imaging for tumor diagnosis and treatment: A comprehensive approach. Discov. Oncol..

[136] Bluethgen C., Van Veen D., Truhn D., Kather J.N., Moor M., Polacin M., Chaudhari A., Frauenfelder T., Langlotz C.P., Krauthammer M. (2025). Agentic Systems in Radiology: Design, Applications, Evaluation, and Challenges. arXiv.

[137] Salehi S., Singh Y., Horst K.K., Hathaway Q.A., Erickson B.J. (2025). Agentic AI and Large Language Models in Radiology: Opportunities and Hallucination Challenges. Bioengineering.

[138] Salehi S., Singh Y., Habibi P., Erickson B.J. (2025). Beyond single systems: How multi-agent AI is reshaping ethics in radiology. Bioengineering.

[139] Han X., Gao X., Qu X., Yu Z. (2025). Multi-agent medical decision consensus matrix system: An intelligent collaborative framework for oncology mdt consultations. arXiv.

[140] Dąbrowicki W., Rusiecki A., Jeleń Ł. (2025). Med-Agent: A Hybrid AI Agent for Multimodal Cancer Diagnosis. Procedia Comput. Sci..

[141] Dietrich N. (2025). Agentic AI in radiology: Emerging potential and unresolved challenges. Br. J. Radiol..

[142] Khosravi B., Rouzrokh P., Akinci D’Antonoli T., Moassefi M., Faghani S., Mansuri A., Bressem K., Tejani A., Gichoya J. (2026). Agentic AI in Radiology: Evolution from Large Language Models to Future Clinical Integration. Radiol. Artif. Intell..

